# Accelerating spiking neural network simulations with PymoNNto and PymoNNtorch

**DOI:** 10.3389/fninf.2024.1331220

**Published:** 2024-02-20

**Authors:** Marius Vieth, Ali Rahimi, Ashena Gorgan Mohammadi, Jochen Triesch, Mohammad Ganjtabesh

**Affiliations:** ^1^Frankfurt Institute for Advanced Studies, Frankfurt am Main, Germany; ^2^Department of Mathematics, Statistics, and Computer Science - College of Science, University of Tehran, Tehran, Iran

**Keywords:** spiking neural network (SNN), comparison, simulator, GPU accelerated, efficient implementation

## Abstract

Spiking neural network simulations are a central tool in Computational Neuroscience, Artificial Intelligence, and Neuromorphic Engineering research. A broad range of simulators and software frameworks for such simulations exist with different target application areas. Among these, PymoNNto is a recent Python-based toolbox for spiking neural network simulations that emphasizes the embedding of custom code in a modular and flexible way. While PymoNNto already supports GPU implementations, its backend relies on NumPy operations. Here we introduce PymoNNtorch, which is natively implemented with PyTorch while retaining PymoNNto's modular design. Furthermore, we demonstrate how changes to the implementations of common network operations in combination with PymoNNtorch's native GPU support can offer speed-up over conventional simulators like NEST, ANNarchy, and Brian 2 in certain situations. Overall, we show how PymoNNto's modular and flexible design in combination with PymoNNtorch's GPU acceleration and optimized indexing operations facilitate research and development of spiking neural networks in the Python programming language.

## 1 Introduction

Computer simulations are a central tool for the scientific study of complex systems. In Neuroscience, owing to the great complexity of the brain and nervous system, computer simulations have become indispensable for scientific progress. Accordingly, various simulators have been designed with a different focus on model generation and analysis.

Brian 2 (Goodman and Brette, 2009; Stimberg et al., 2019) stands out because it allows for representing neural dynamics directly as differential equations with physical units. NEST (Gewaltig and Diesmann, 2007) excels in running large-scale simulations (Jordan et al., 2018) on computing clusters and serves as a reference implementation for neuromorphic hardware (van Albada et al., 2018) and NeuronGPU is currently integrated into the NEST initiative as NEST GPU to provide GPU acceleration (Golosio et al., 2021). BindsNET (Hazan et al., 2018), SpikeJelly (Fang et al., 2023), Norse (Pehle and Pedersen, 2021), PySNN (Büller, 2019), Rockpool (Muir et al., 2023), cuSNN (Paredes-Valles et al., 2020), and snnTorch (Eshraghian et al., 2023) facilitate the use of spiking neural networks for machine learning-oriented experiments.

NEURON (Carnevale and Hines, 2006) and Genesis (Bower et al., 2003) focus on detailed neuron models with intrinsic compartments. On the other hand, ANNarchy (Vitay et al., 2015) offers the advantage of supporting rate coding models. Some other simulators, such as CARLSim (Nageswaran et al., 2009; Niedermeier et al., 2022), prioritize efficient and machine-oriented implementations, allowing models to run on robotics hardware. Libraries like Nengo (Bekolay et al., 2014) facilitate chaining up trained spiking sub-networks to approximate different mathematical functions. There are simulators available for designing complex neuronal networks and the nervous system of whole organism in 3D, such as NeuroConstruct (Ghahremani et al., 2022). Some of these simulators provide native GPU support, for example GENN (Yavuz et al., 2016) and NeMo (Fidjeland, 2014), while others offer extensions like Brian2Cuda (Alevi et al., 2022) or Brian2GeNN (Stimberg et al., 2020). Efforts have also been made to streamline model development with PyNN (Davison et al., 2009) or NeuroML (Gleeson et al., 2010) by providing uniform languages for model descriptions independent of the underlying simulator.

Among these simulators, PymoNNto (Vieth et al., 2021) has a simple skeleton while being modular and extendable. Its simplicity makes it unchallenging and lets researchers concentrate on the model, while its modularity and extendability allow for novel and unconventional model design. Although the flexibility of PymoNNto allows for the use of hardware accelerators such as GPUs, this requires hardware-specific implementation of modules. Hence, we here introduce PymoNNtorch, a PyTorch-adapted version of PymoNNto. Using PyTorch (Paszke et al., 2019) instead of Numpy (Harris et al., 2020) as the tensor-computing backend allows utilizing the same module on various computing hardware supported by PyTorch and, consequently, precludes repetitive implementation.

When deciding among different simulators for a particular project, computation speed is always a central concern. Even though hardware accelerators can significantly decrease the simulation time, the impact of an efficient implementation is substantial. As PymoNNto(rch) only provides a framework, its efficiency heavily depends on the user implementation of neural, synaptic, and sundry other dynamics. Thus, in this article, we present some simple but also subtle methods to efficiently implement operations commonly used in spiking neural network (SNN) simulations. The changes may appear minor, but we show that they can yield enormous speed-ups. In particular, our results demonstrate that efficient implementations can speed up simulations by over three orders of magnitude compared to naive implementations.

Furthermore, we have developed two representative models and conducted an analysis of their respective efficiencies for different versions of the NEST, ANNarchy and Brian 2 simulators. The first model combines Leaky Integrate and Fire (LIF) neurons with a simple step-wise STDP function (see below). Such a learning rule is also quite common and similar concepts can be found in various works about the SORN model Lazar et al. (2009) or other works like Masquelier and Thorpe (2007), Rolls (2010), Stocco et al. (2010), Soures et al. (2017), Tomasello et al. (2018), or Gautam and Kohno (2021) to name some examples. The second one is a more conventional model consisting of the Izhikevich neuron model combined with a standard widely used trace STDP rule (Song et al., 2000; Cohen et al., 2007; Galluppi et al., 2015; Qiao et al., 2019).

The results of this analysis show that PymoNNto(rch), if the user implements the behaviors carefully, can outperform comparing simulators, especially with a simpler Hebbian learning rule and dense connectivity. Moreover, using hardware accelerators, such as a GPU, can considerably accentuate the improvements. Such custom, fast and easily expandable network simulations in combination with novel learning and stability mechanisms hold a great potential advancing the field of computational neuroscience.

## 2 Methods

Due to the mutual underlying structure between PymoNNto and PymoNNtorch, we only present the essential parts and highlight the differences. For more details, we recommend the original PymoNNto publication (Vieth et al., 2021) and its online documentation.^1^ Afterwards, we explain how a model can be created using PymoNNto(rch) and how it internally processes the simulation. Then, we investigate faster implementations for operations prevalent in spiking neural networks. The source code for PymoNNto^2^ and PymoNNtorch^3^ is publicly available under the MIT license on their corresponding GitHub pages. Documentations and tutorials can be found on the ReadtheDocs pages.^1^^,^^4^

### 2.1 Architecture

Any simulator has restrictions on the configuration of the simulation, and PymoNNto(rch) is no exception. As it is common in computational neuroscience, PymoNNto(rch) adopts a discrete time-based simulation framework, where simulation time discretely advances and the system state is updated according to the last time step. Distinct from ordinary, PymoNNto(rch) separates the model components from their dynamics. This separation allows expressing the structure of a model, abstract definitions of components and their relations to other components while keeping behavior parts simple and easily modifiable, hence facilitating experimentation.

**Figure 1:**
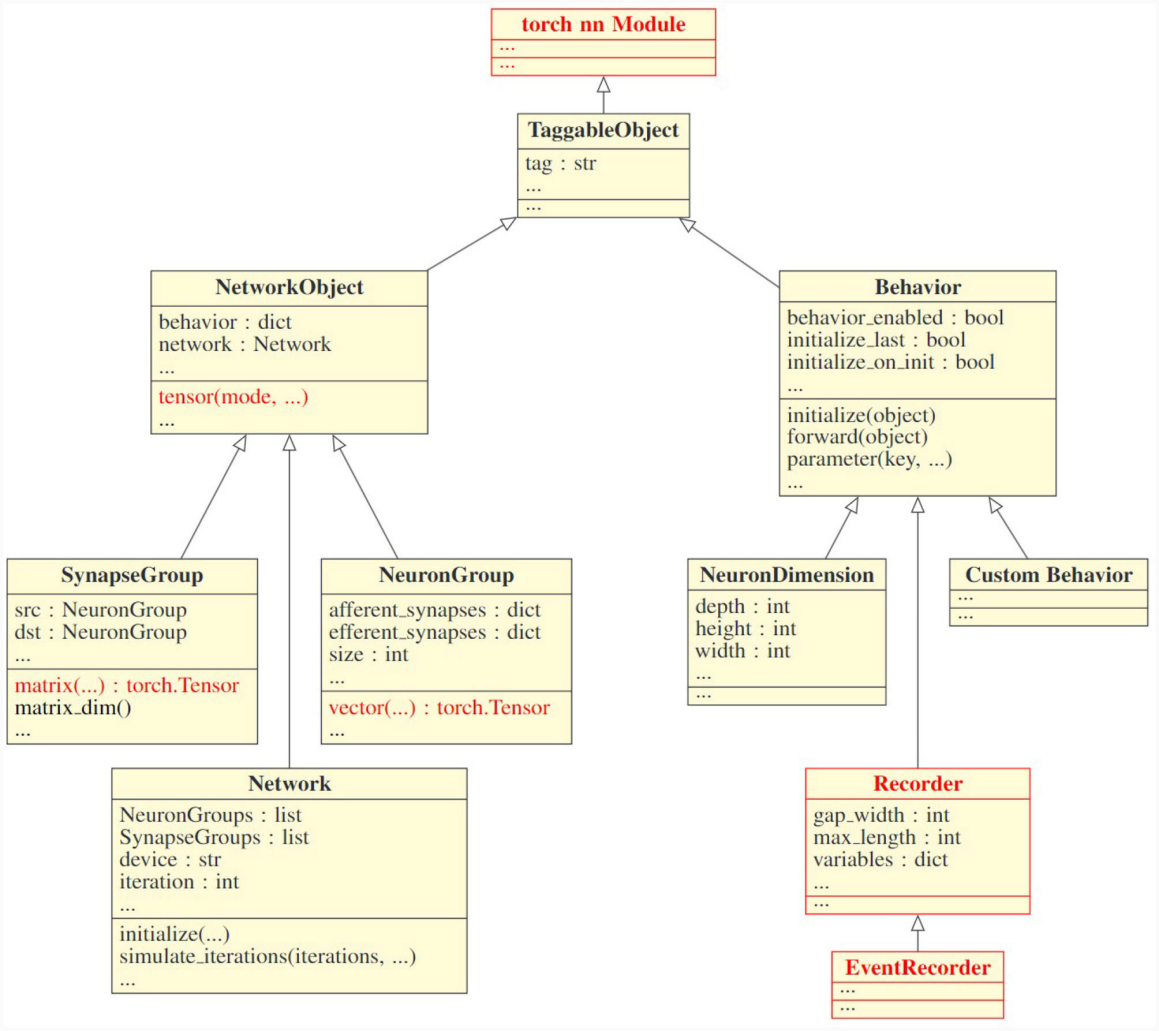
Class inheritance structure of PymoNNtorch. This UML diagram only represents PymoNNtorch's fundamental classes. The arrows indicate the inheritance relationship. The classes and functions in red are what distinguishes PymoNNtorch from PymoNNto.

Figure 1 depicts the internal design of PymoNNtorch. Each class derived from NetworkObject is an abstract component, and each class derived from Behavior is responsible for dynamics, where each component could accept multiple behaviors. Behaviors attached to a component (1) have a unique integer *key* responsible for the order of execution, (2) can modify the components's properties, and (3) apply dynamics during the simulation.

Just as in PymoNNto, PymoNNtorch has four integral object classes as follows:

1. NeuronGroup:
  It is reasonable to consider neurons with similar characteristics as a single homogeneous population. This approach leads to a more efficient execution while simultaneously simplifies the model. A NeuronGroup is a component designed to serve as the structure for a population of neurons. It holds two dictionaries for the afferent and efferent synapses it is connected to. A NeuronGroup also has an auxiliary function, entitled *vector*, to initialize population-size tensors. These tensors are suitable for storing various properties such as membrane potential and firing state. During the simulation, a NeuronGroup does not execute anything itself. Instead, the attached behaviors perform the desired instructions.
2. SynapseGroup:
  Similarly, to expedite synapse creation and synaptic calculation, SynapseGroup has been designed. It holds the connected pre- and post-synaptic populations. The auxiliary function *matrix* creates a matrix to represent the synaptic connections from the pre-synaptic population to the post-synaptic population. Like any other component, without any behavior attached to it, a SynapseGroup is inactive.
3. Network:
  While the structure of neurons and synapses in the brain are inseparable from their functionalities, the same does not hold for a simulation on conventional computer architectures. A central process should be responsible for holding the structure of the model and executing its instructions. Resultantly, the Network class has been designed. Although a Network is still a component and can have its own network-wide behaviors, it possesses all the components and their associated behaviors. A network is also responsible for advancing the simulation and managing the order in which behaviors are executed. Changing the hardware on which the simulation executes, is as easy as creating the network with a desired device as an argument.
4. Behavior:
  In a complex system, components can be distinguished by the dynamics they act upon. Breaking down the dynamics into smaller and simpler dynamics and replicating them is essential to apprehending the components and the whole system. Therefore, the Behavior class is designed to be responsible for adding dynamics to the components it attaches to. The crux of the simulation is to execute these behaviors. In order to generate a new behavior, a derived class from Behavior should be made and subsequently, the *initialize* and *forward* methods should be overloaded. The *initialize* method is responsible for creating variables for the behavior itself as well as the attached component and the *forward* method applies the dynamics to the variables during the simulation. Both methods receive the component they are attached to as an argument. In Section 2.3, we present guidelines on how to implement behaviors in an efficient way.

All components and behaviors inherit from the TaggableObject, which allows for conveniently finding any component, behavior, or tagged belongings within them. Moreover, what makes PymoNNtorch different from PymoNNto is that all the components and behaviors are based on the torch.nn.Module, which facilitates working with the tensors in PyTorch. All the auxiliary functions like *vector(), matrix()*, and their root function *tensor()* return PyTorch tensors, and they internally manage the datatype and the device of the tensor. Also, Recorders accumulate the state of variables in the device where the network resides.

Although PymoNNtorch and PymoNNto are designed to simulate spiking neural networks, they are suitable for simulating any graph-like structure as long as the computation of each component can be performed on local variables only. One can see NeuronGroups as nodes and SynapseGroups as edges of a graph. Additionally, a novel structure can be easily made by inheriting from the NetworkObject class.

### 2.2 Simulation process

**Figure 2:**
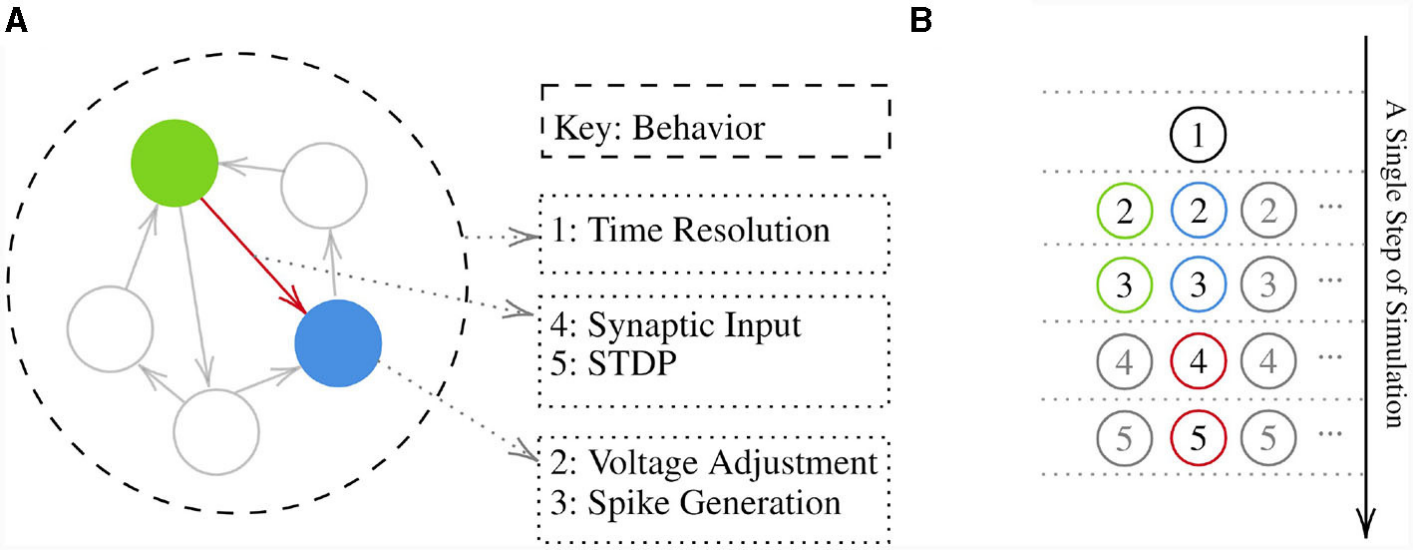
**(A)** Visualization of a hypothetical network with multiple neuron groups shown by circles and arrows representing synapse groups between them. Behaviors of a neuron group, a synapse group, and the network with their corresponding key are shown on the right. **(B)** Shows a pipeline of behaviors execution in a single step of simulation on the sample network presented. Note that numbers inside the circles are the keys that behaviors are attached via them to a component. These keys determine the execution order of behaviors with regard to the component holding them.

To make a model, first a Network instance should be created. This instance will contain all the NeuronGroups and SynapseGroups created later. It also collects all the behaviors and organizes them based on the provided priority keys. Creating a component requires implementing new behaviors or reutilizing already implemented ones and attaching them to that component. Once all components have been created, the *initialize* method of the network should be called to ask each behavior to run its own *initialize* method to prepare their required variables and set the initial conditions on them. Then the *simulate_iterations* method of the network should be called to make a loop to execute all the behaviors' *forward* method repeatedly. Note that the priority keys associated with behaviors are not local but rather global. Figure 2 depicts a sample network and the execution order of its behaviors.

### 2.3 Fast network implementation

In the preceding section, we explored the fundamental components of PymoNNto(rch) and observed that the behavior modules could contain arbitrary Python code from various libraries, including NumPy and PyTorch. In this section, we demonstrate how basic SNN operations can be improved and efficiently implemented using NumPy and PyTorch functions. These optimized operations can be effectively employed to accelerate the performance of a wide range of custom network models. We will incorporate these enhancements into our implementations in both PymoNNto and PymoNNtorch, subsequently subjecting them to comparison against other simulators.

To facilitate a more precise comparison of the individual improvements, we employ timing measurements to gauge the computational cost of each operation. These measurements represent the average execution time based on 1,000 independent runs and are presented alongside the respective code lines. All measurements in this artile were conducted on an Amazon AWS EC2 instance. The details regarding the software and hardware are described in the Supplementary material. All the code examples, experiments, and results can be found on GitHub.^5^

In the following examples, we will begin by presenting a naive implementation of desired calculation (depicted in the red code block) and then demonstrate how it can be improved (shown in the green code block). We assume an SNN simulation running in discrete time steps, two groups of neurons and a group of dense synapses connecting them. To represent the firing states of these neuron groups, we employ binary spike vectors labeled as “src” and “dst,” containing 5,000 and 10,000 neurons, respectively. The synapses are characterized by a dense weight matrix denoted as *W*1, encompassing a total of 50 million synapses.

Initially, we set the variables with the following parameters:


S = 5000
D = 10000
  
‘‘Numpy’’
src = np.random.rand(S)  <  0.01          # 1% spikes
dst = np.random.rand(D)  <  0.01          # 1% spikes
W1 = np.random.rand(D, S)                 # dense weight matrix (D×S)
  
‘‘PyTorch’’                               # d = ‘cpu’ or ‘gpu’
src = torch.rand(S, device=d)  <  0.01
dst = torch.rand(D, device=d)  <  0.01
W1 = torch.rand(D, S, device=d)


#### 2.3.1 Synapse operation

The transmission of information across synapses is a vital aspect of neural networks. The most basic method involves employing a straightforward matrix-vector product, which can be implemented as follows:


‘‘Numpy’’
W1.dot(src)                                 # 14.8ms      (naive) *Baseline
  
‘‘PyTorch’’
torch.tensordot(W1, src, dims=([1],[0]))    # CPU: 15.1ms, GPU: 1.7ms


However, considering that the spike vector comprises only zeros and ones, we can sidestep resource-intensive multiplications and achieve equivalent results through indexing and summation. This optimization alone results in more than a roughly six-fold or higher increase in speed:


‘‘Numpy’’
np.sum(W1[:, src], axis=1)      # 2.2ms (6.6×)
  
‘‘PyTorch’’
torch.sum(W1[:, src], dim=1)    # CPU:  4.1ms (3.5×), GPU: 0.3ms (39.2×)


#### 2.3.2 Weight matrix storage

In the two previous code blocks, the weight matrix *W*1 possesses dimensions D×S, where *D* represents the size of the destination neuron group, and *S* denotes the size of the source neuron group. While this notation aligns with standard mathematical conventions and obviates the necessity for an additional transpose operation during synaptic transmission simulation, it can be computationally inefficient from a practical standpoint, as most memory access is not aligned with the row-major convention utilized for storing matrix elements in memory.

**Figure 3:**
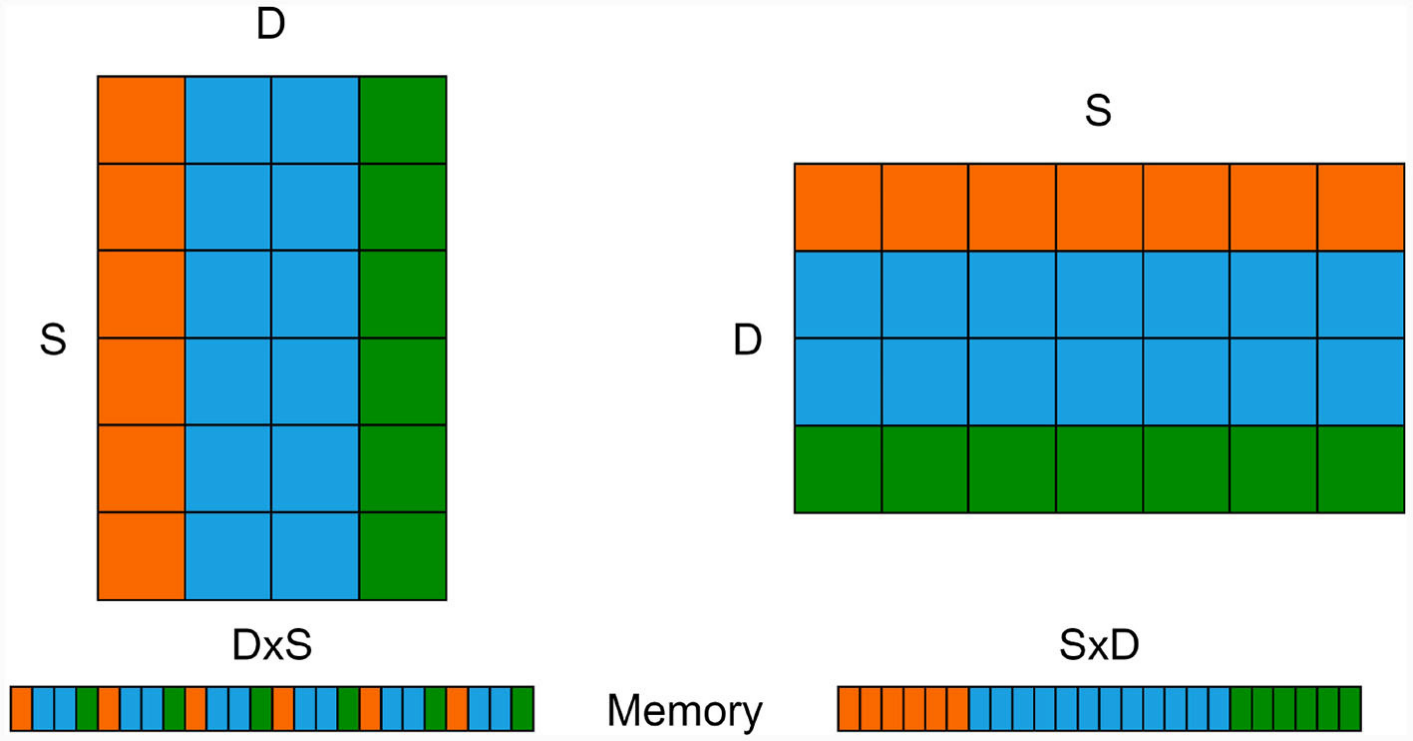
S×D weight matrix **(left)** and its transposed D×S version **(right)** along with their corresponding memory allocations **(bottom)**. The orange and green rows/columns signify the afferent weights of two exemplar neurons, which must be combined to calculate the input for each neuron.

Figure 3 provides a visual representation of the memory storage for both D×S and S×D matrices. In this illustration, the orange and green “bars” symbolize the input synapses for two exemplar neurons. To obtain the cumulative input to a single neuron, we must sum the weights of active inputs within a single bar. However, when using the “intuitive” D×S synapse matrix approach, the values within a single bar are dispersed across the memory block which is not cache-friendly.

Switching the dimensions such that all values within the same “bar” are stored in a contiguous memory section enables the CPU/GPU to request an entire memory block rather than individual values. This optimization can significantly expedite processing. Modifying the code accordingly, as demonstrated below, yields a remarkable speed-up of up to 38-fold compared to the baseline version:


''NumPy''
W2 = np.random.rand(S, D)       # dense weight matrix       (SxD)
np.sum(W2[src] , axis=0)        # 0.58ms (25×)
  
''PyTorch''
W2 = torch.rand(S, D, device=d)
torch.sum(W2[s], dim=0)         # CPU: 0.38ms (38×), GPU: 0.11ms (130×)


#### 2.3.3 Synaptic plasticity

SNNs are often trained using various forms of unsupervised Hebbian-like learning rules that take into account the activities of pre- and post-synaptic neurons. A special rule is spike timing-dependent plasticity (STDP) (Markram et al., 1997), which considers the relative timing of pre- and post-synaptic spikes.

**Figure 4:**
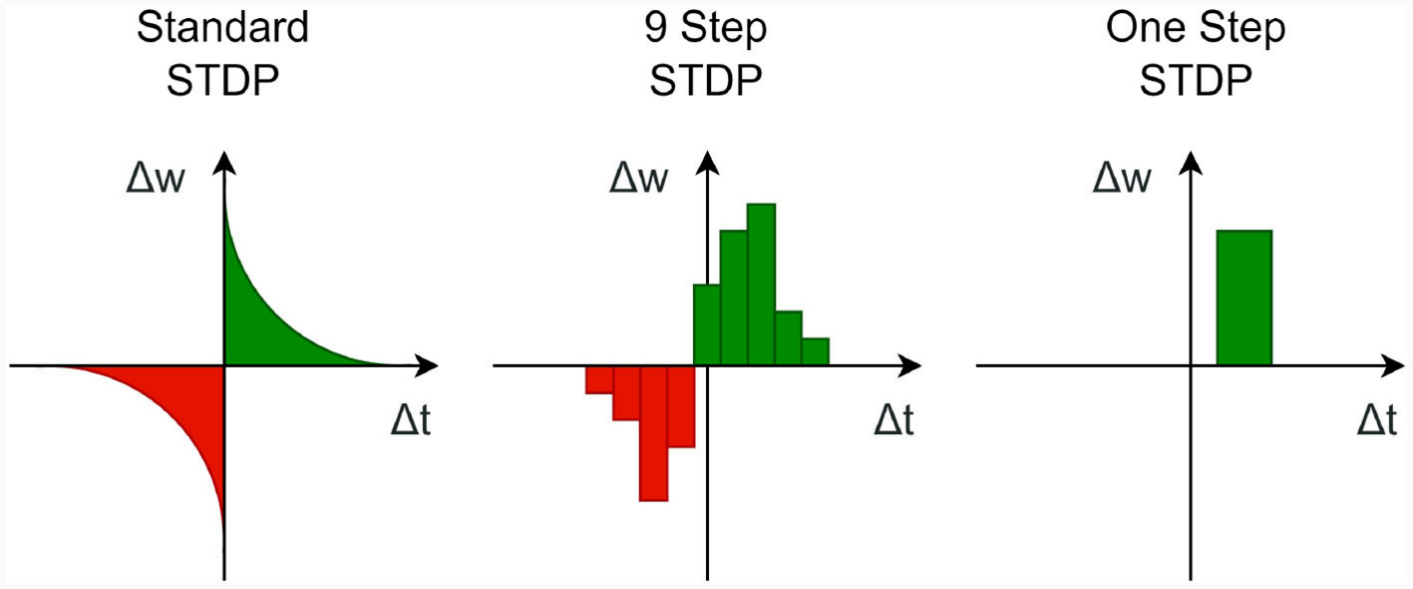
Three types of STDP functions, with different levels of detail.

Numerous methods exist for implementing these kind of learning rules. In this context, we consider the simple approach of correlating (possibly time-shifted) pre- and post-synaptic spike trains as used in Hopfield-type networks (Hopfield, 1982) or SORNs (Lazar et al., 2009) (see Figure 4 “One Step”).

In the subsequent discussions, we work with both D×S and S×D matrices. However, in contrast to the previous examples, this alteration has minimal impact on the performance of the improved version. This is primarily related to the fact that no operations over entire columns or rows are required in this particular context.

We consider spike vectors labeled “src” and “dst” for representing the (potentially time-shifted) pre- and post-synaptic activities, respectively. The most straightforward approach to compute the weight changes (disregarding learning rates, etc.) is by utilizing a basic outer product of these vectors, implemented as follows:


''NumPy''
W1 += dst[:,None] * src[None,:]     # 74ms (naive) *Baseline
W2 += src[:,None] * dst[None,:]     # 116ms
''or''
W1[dst[:,None] * src[None,:]] += 1  # 39ms
W2[src[:,None] * dst[None,:]] += 1  # 81ms
  
''PyTorch''
W1 += dst[:,None] * src[None,:]     # CPU: 220ms, GPU: 3.9ms
W2 += src[:,None] * dst[None,:]     # CPU: 220ms, GPU: 3.9ms
''or''
W1[dst[:,None] * src[None,:]] += 1  # CPU: 149ms, GPU: 3.3ms
W2[src[:,None] * dst[None,:]] += 1  # CPU: 148ms, GPU: 3.3ms


This function generates a new D×S weight matrix that is either added to the current weights or used for indexing. However, both of these approaches are highly inefficient. For instance, consider a weight matrix *W*1 containing 5,000 × 10,000 elements; This matrix amounts to 50 million multiplications, where most of them involve at least one factor that is zero, since typically only a small number of pre- and post-synaptic neurons are expected to be active. Remarkably, by employing sparse indexing, we can accelerate the computation by approximately three orders of magnitude, achieving the following significant improvement:


''NumPy''
W1[np.ix_(dst, src)] += 1 # 0.0694ms (1078×)
W2[np.ix_(src, dst)] += 1 # 0.0669ms (1118×)
  
''PyTorch''
W1_index = (torch.where(dst)[0].view(-1,1), torch.where(src)[0].view(1,-1))
W1[W1_index] += 1 # CPU: 0.1530ms (489×), GPU: 0.1714ms (436×)
W2_index = (torch.where(src)[0].view(-1,1), torch.where
(dst)[0].view(1,-1))
W2[W2_index] += 1 # CPU: 0.1522ms (491×), GPU: 0.1715ms (436×)


This version selectively modifies those elements in the weight matrix that require updates while preserving the rest of the elements. This is accomplished using the np.ix_(a, b) function, which computes a sparse mesh containing only the indices of the values that need modification. In PyTorch, a similar outcome can be achieved using the “where” function.

The sparse indexing technique can indeed be extended to implement a more intricate STDP process. For instance, to create an STDP process, like the one depicted in Figure 4 “9 Step,” one can repeat the previously mentioned computation multiple times for different time steps. Furthermore, if a wider STDP window is required, it may be practical to bundle the spike vectors from multiple time steps together and execute the STDP process only at specific intervals. This approach can help to manage computational complexity while accommodating broader temporal windows for plasticity modulation in neural networks.

#### 2.3.4 Weight clipping and normalization

It is frequently desirable to limit the growth of synaptic weights to prevent potential instabilities. The simplest method to achieve this is through utilizing a hard-bound over weights known as weight clipping. Incorporating weight clipping into the previous example yields the following modification:


mask = np.ix_(dst, src)
W1[mask] += 1
W1[mask] = np.clip(W[mask], W_min, W_max)


To reduce unnecessary computations, we exclusively apply clipping to the synaptic weights that have been modified (as demonstrated in the previous example) to ensure that they remain within the specified range [*W*_min_, *W*_max_]. Additionally, we continue to leverage our sparse mesh for efficiency in this context.

Another widely used mechanism is neuron-wise weight normalization, as described in Elliott (2003). This normalization ensures that the total amount of afferent synaptic weights remains constant for each neuron. While this operation can be computationally expensive when executed at every time step, it is often sufficient to perform it at specific intervals, reducing the overall computational load:


if iteration % 100 == 0:            # normalize only every 100 iterations
    W /= np.sum(W, axis=1)[:, None] # afferent (D×S)
    W /= np.sum(W, axis=0)          # efferent (D×S)


#### 2.3.5 Smart choice of data types and sparsity

Lowering the precision of computations is another straightforward method to enhance the speed of vector and matrix operations. This can be accomplished by adjusting the “dtype” parameter:


# synapse operation with:
dtype = np.float64 # 0.55ms (default)  *Baseline
dtype = np.float32 # 0.29ms (1.88×)
dtype = np.float16 # 3.65ms (0.15×) !


As a general rule, lower precision is often expected to yield faster computation times. However, it is essential to be mindful of exceptions, such as the last line of the previous example, where not all CPUs support accelerated processing of smaller data types like 16-bit floating point precision.

Whether a lower precision is acceptable heavily depends on the chosen model, its step size and the implemented modules. However there are many cases in which the more than 8 million mantissa (23 bit) and the 256 (8 bit) exponential states of float32 are more than enough to represent properties of simplified neuron models.

It is worth noting that specialized “sparse matrix” data types, such as those available in SciPy, can be used with PymoNNto(rch) but they may not always lead to speed improvements. These matrices employ list-like data structures that can be relatively slow in many scenarios. Additionally, the optimization methods presented in this article do not necessarily apply to various representations of a sparse matrix.

Nevertheless, a simple example with sparse matrices is provided in the Supplementary material. In this implementation, the break-even point at which sparse matrices are faster than the optimized dense approach is below one percent connectivity. Even though we did not delve into sparse-specific matrix operation optimization approaches, since any code can be packed into the behavior modules, further optimization approaches for sparse representations are possible.

The main advantage of a sparse matrix implementation lies in its reduced memory usage. Nevertheless, the matrix *W* with 32-bit floating point numbers from above and 50 million elements only consumes 200 MB of memory. We also investigated whether the processing time changes when we fill the matrix with zeros to simulate sparse connectivity. We noted no noticeable differences on the employed hardware, indicating that multiplications with zeros or ones are not handled differently.

If the neuron population grows to a size where an all-to-all connectivity may become impractical, PymoNNto(rch) provides the option to either create multiple smaller neuron groups or to divide a large neuron group with masks into smaller subgroups. These smaller groups can then be connected through multiple dense synapse groups, resulting in potential savings in both memory and processing power. Without these subgroups, the all-to-all connection scheme may demand excessive memory for large neuron populations, highlighting a limitation in our current approach.

### 2.4 Implementation example

Since behaviors in PymoNNto(rch) incorporate regular Python codes, it is possible to seamlessly integrate the previously discussed examples into the corresponding behavior modules. The codes under the Numpy section are compatible with PymoNNto and the PyTorch sections are compatible with PymoNNtorch. By changing the variable names we can directly put the previous operations into our modules. Here is an example of what the STDP module might look like in PymoNNto(rch):


class STDP(Behavior):
  
    def initialize(self, neurons):
        self.learning_rate = self.parameter('learning_rate')
  
    def iteration(self, neurons): # def forward(...): in PymoNNtorch
        for s in neurons.synapses(afferent):
            mask = np.ix_(s.src.spikesOld, s.dst.spikes)
            s.W[mask] += self.learning_rate
            s.W[mask] = np.clip(s.W[mask], 0.0, 1.0)


As it can be seen, the last three lines are just copied into the scaffold and we added a “learning_rate” parameter. Once this STDP module is defined, it can be attached to any NeuronGroup, and the for-loop mechanism ensures that the module can also efficiently process multiple connected SynapseGroups when necessary.

Note that the overhead introduced by PymoNNto(rch) to the Python/NumPy/PyTorch code is practically negligible (refer to Supplementary Figures S1, S2). At each time-step, PymoNNto(rch) iterates over a list with all the attached modules, already sorted in the correct order. For each module, it executes a simple function call, which takes approximately one microsecond on the utilized machine.

## 3 Results

**Figure 5:**
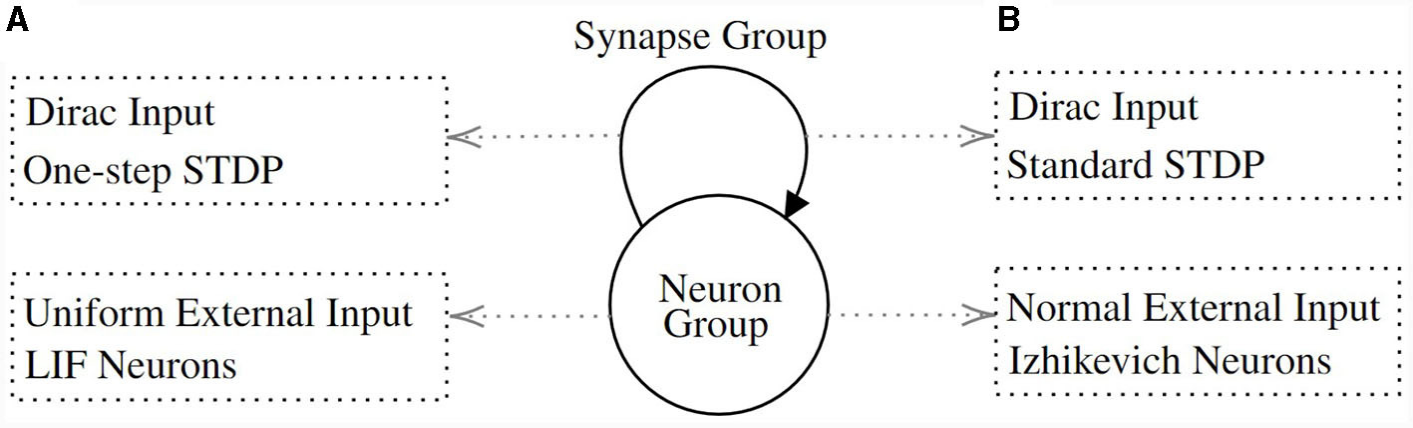
The model structure of experiments. It consists of a neuron group and a synapse group connecting the neurons within the group. **(A)** The dynamics for the first and second experiments. The neurons follow the LIF dynamics and they receive a uniformly distributed external input. The synapse is fully connected. It acts as a Dirac function and its weights are updated with the One-Step STDP function. **(B)** The dynamics for the third experiment. The neurons feature the Izhikevich dynamics and receive a normally distributed external input. Also, synapse weights are updated with the standard STDP function (Figure 4 “Standard”).

In this section, we seek to compare various network implementations across different simulators. We conducted three sets of experiments. Firstly, we compare the optimized and naive versions of PymoNNto(rch). Secondly, we implement an equivalent network (Figure 5A) in Brian 2, NEST and ANNarchy and measure their respective performances. Thirdly, we utilize a popular network model that combines an Izhikevich neuron model (Izhikevich, 2003) (Figure 5B) with standard STDP (as shown in Figure 4 “Standard”) and present the results from different simulators side by side. We selected these models because they establish a common baseline for a wide range of existing learning models found in current literature. To enhance clarity, we minimize the number of parameters in the models and keep them as simple as possible while providing synapse and plasticity operations. The models do not contain additional homeostatic mechanisms, therefore, they rely solely on strong external noise to drive the network activity and to stabilize the spike rate. Because of the relatively weak synapses, the models exhibit similar firing rates across different population sizes (Supplementary Figures S5, S6). We also ensured that the models generate comparable population activity (see Supplementary Figures S2, S4) and that there are no significant rounding errors caused by the involved parameters, such as step-size and data-types. In all models, the main computational load is caused by the synapse operations and STDP, especially by the standard STDP rule (see Supplementary Figures S1, S3). All implementation details, individual measurements of the employed modules and additional information can be found in the Supplementary material.

### 3.1 Optimized vs. naive approach

In the Methods section, we have presented the details of different approaches for implementing networks with greater efficiency.

In this section, we combine several elements, including basic leaky integrate-and-fire (LIF) neurons with random fluctuations, fast synapse operations, One-Step STDP, transposed matrices, weight clipping, and the use of float32 data types, into a single “optimized” network model. We then proceed to compare this optimized model to a “naive” implementation that lacks these efficiency enhancements.

**Figure 6:**
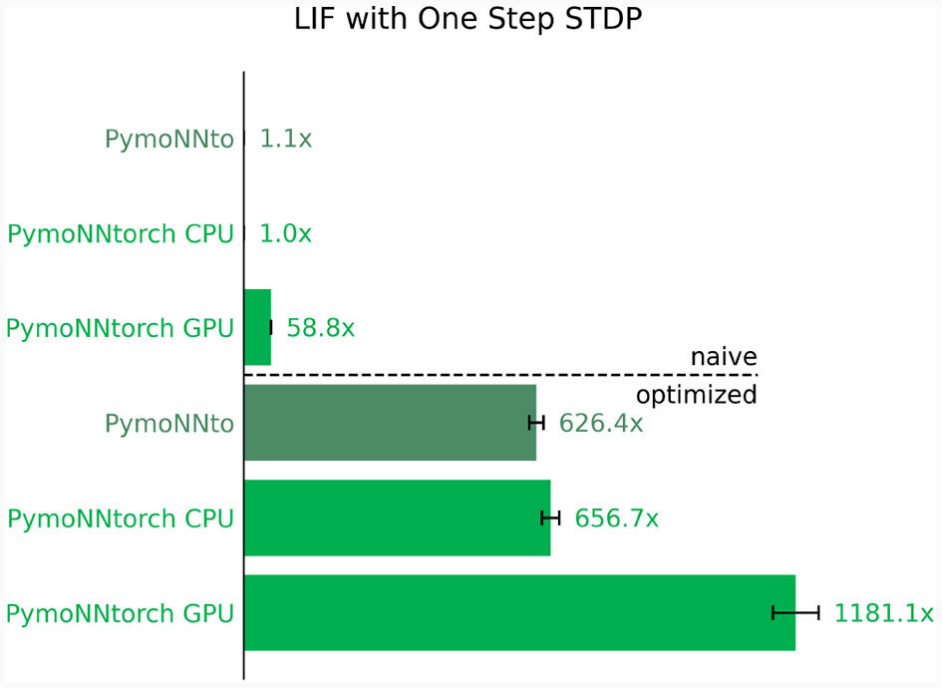
Ratio of improvements in processing speed compared to the slowest version. Naive implementation **(Top)** vs. optimized implementation **(Bottom)** for the same network. Averaged over 10 independent runs for a duration of 300 time steps; The ratios indicating how many times faster the individual simulation is compared to the slowest one.

The results are illustrated in Figure 6, where the top represents the optimized implementations and the bottom displays the naive implementations. It's worth noting that these straightforward modifications have led to a substantial speed-up of over three orders of magnitude. Furthermore, we observe that in both implementations, the PymoNNtorch GPU version outperforms the CPU version. Supplementary Table S8 details the effectiveness of individual optimization methods on total simulation time.

Nonetheless, the speed remains relatively comparable between the optimized CPU and GPU implementations, and there are scenarios in which optimizing operations is not as straightforward. For instance, consider a rate-based network that relies on analog values instead of spikes, rendering indexing less effective. Additionally, operations like weight normalization demand dense computations. Therefore, the performance advantage of using a GPU is highly contingent on the specific network architecture in use.

### 3.2 Simulator comparison

Next, we proceed to compare the performance of various simulators with each other. To do this, we utilize the previously discussed simple optimized network of LIF neurons with One-Step STDP as well as a network of Izhikevich neurons with standard STDP.

To perform the comparison, we have examined a total of nine different simulators, including their variations. These incorporate the three PymoNNto(rch) versions, which remain consistent with the ones used in the previous experiment. Additionally, we incorporate Brian 2, which can also be compiled into C++ code using the “cpp_standalone” package or take advantage of GPU acceleration with the “cuda_standalone” parameter (Alevi et al., 2022), both of which can enhance its efficiency. We also conducted experiments on the ANNarchy simulator and tested the CPU as well as the CUDA GPU version. As for the NEST simulator, we have employed a native implementation for the network with LIF neurons, and for the network with the Izhikevich neurons, we have utilized the PyNN interface. Initially, we intended to use PyNN for all our comparisons, however, it proved to be limiting in terms of required custom implementations and the C++/Cuda optimizations.

It is important to note that, when using the same parameters and equations, the native NEST implementation consistently generates slightly fewer spikes in comparison to the other simulators. This leads to a slight reduction in its simulation time since it needs to compute fewer events. The NEST simulator is also the only one which only uses the double precision values while all other simulators are set up to use single precision. The reason for this is that there seems to be no setting to change the default datatype of NEST. This, however, is not the reason for its slightly lower spike count because the other simulators do not show this behavior when they are set up to use float64 datatype.

**Figure 7:**
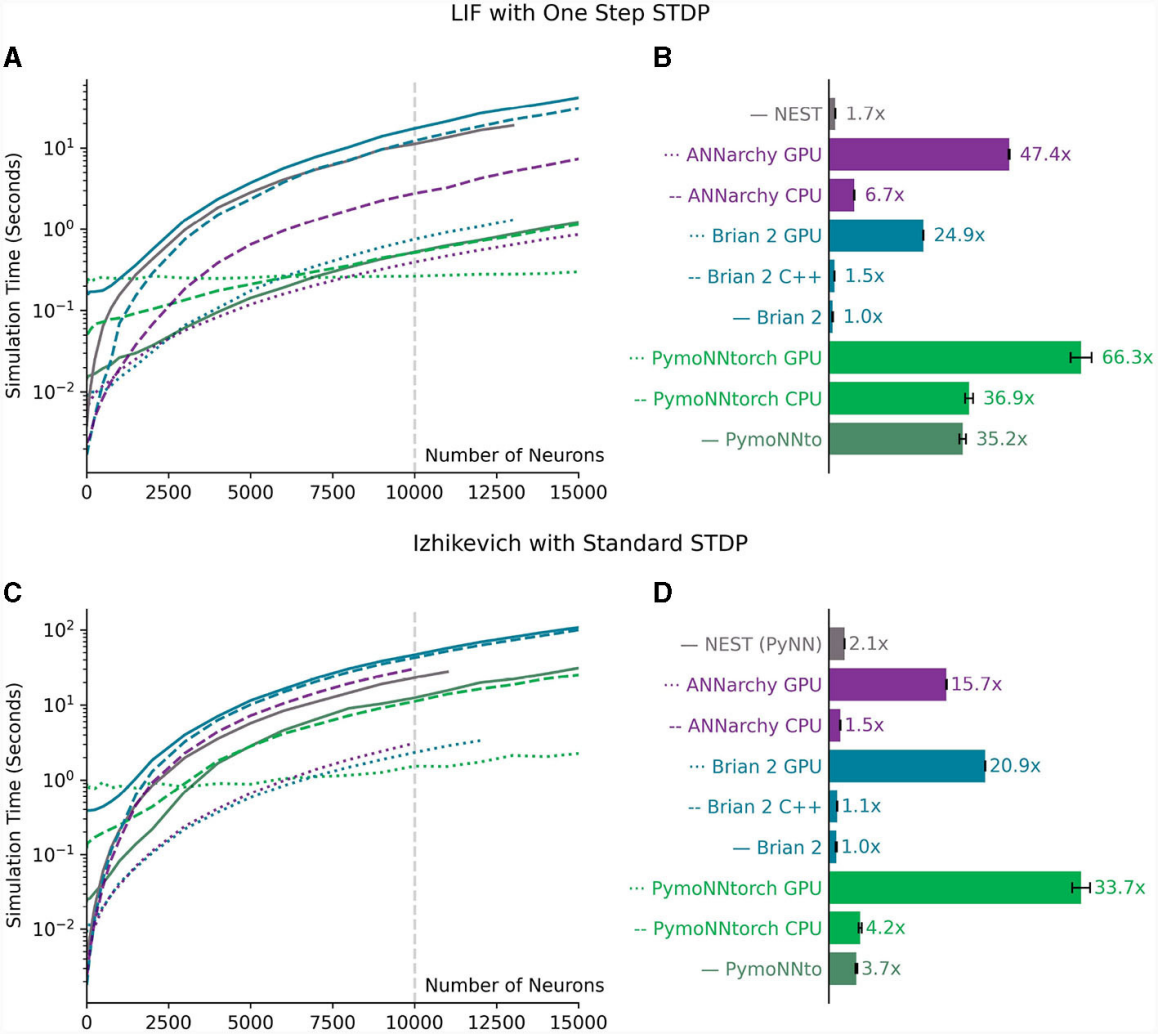
**(A, B)** Measurements for a network of LIF neurons from Figure 6. **(A)** Measured simulation time (log scale) for different network sizes. **(B)** The ratio of processing speed-ups for different simulators compared to the slowest one (300 time steps averaged over 10 independent runs for a network of 10,000 neurons). **(C, D)** Measurements for a network of Izhikevich neurons with standard STDP shown in Figure 4 “Standard.” **(C)** Measured simulation time (log scale) for different network sizes. **(D)** The ratio of processing speed-ups for different simulators compared to the slowest one (300 time steps averaged over 10 independent runs for a network of 10,000 neurons). Error bars in **(B, D)** indicate standard deviations. The gray vertical lines in **(A, C)** indicate the network sizes used in **(B, D)** respectively.

The results have been visually represented in Figure 7. Notably, PymoNNto(rch) consistently exhibits superior performance for larger networks, particularly when employing One-Step STDP rule (Figures 7A, B). In these cases, the PymoNNtorch GPU version demonstrates a 39-fold (66.3/1.7) speed-up advantage over the NEST simulation. It is also between 2.6 (66.3/24.9) to 66 times faster compared to the various versions of Brian 2. The CPU version of PymoNNtorch is 5.5 (36.9/6.7) times faster than the CPU version of ANNarchy and on the GPU, PymoNNtorch is 39% faster than ANNarchy.

Even in the case of the Izhikevich version (Figures 7C, D), we observe substantial performance improvements, despite the absence of optimizations for the employed standard STDP rule. In this context, both of PymoNNto(rch)'s CPU versions outperform the other CPU versions and the same is the case for PymoNNtorch's GPU version, which achieves a notable 61% increase in speed compared to Brian 2's GPU version, which came out second.

The logarithmic plots in Figures 7A, C illustrate that on small-size networks, simulators that utilize compilers namely NEST, Brian 2 GPU, Brian 2 C++ and ANNarchy perform faster. Also on small-size networks, the framework overhead of Pytorch causes PymoNNtorch to perform slower than PymoNNto. However, as the number of neurons increases, the speed improvements of PymoNNto(rch) become more pronounced. It becomes evident that simulations can greatly benefit from PymoNNto(rch)'s approach, both in processing and efficient memory usage. The detailed simulation times of Figures 7A, C can be found in the Supplementary Tables S10, S13, respectively.

## 4 Discussion

In this article, we have introduced PymoNNtorch and showcased its optimizations for PyTorch in comparison to PymoNNto, highlighting its native GPU support to accelerate operations for spiking neural network simulations. Additionally, we have outlined a set of techniques designed in both NumPy and PyTorch to efficiently compute commonly used operations in SNNs. Through the utilization of GPUs, transposed synapse matrices, indexed summations instead of vector products, sparse meshes instead of matrix multiplications, and the adoption of smaller data types, we have successfully demonstrated substantial speed enhancements of PymoNNto(rch) in our study.

The techniques that leverage the binary states of SNNs are versatile and can be applied to various SNN models without the need for additional libraries or hardware support. In addition, we have demonstrated that the optimized NumPy and PyTorch implementations can be seamlessly integrated into PymoNNto(rch) modules, which helps structure the code, simplifies further extensions and takes advantage of the PymoNNto(rch) ecosystem. Comparing the optimized version to the naive implementation, we have achieved an acceleration of over three orders of magnitude.

Furthermore, our results have shown that PymoNNto(rch) can significantly outperform optimized simulators such as Brian 2 (default, C++, and GPU versions), ANNarchy and NEST in both exemplar networks. PymoNNto(rch) offers substantial advantages, particularly for researchers working with custom and experimental network models that deviate from traditional implementations. These benefits include significant speed improvements of up to 66-fold, user-friendliness, and a wide range of options for implementing models. This may be particularly useful in neuromorphic engineering contexts, where the goal is not to faithfully model a particular biological system, but to explore a wider range of network architectures, learning mechanisms, and hardware implementations to efficiently solve particular problems.

The choice between PymoNNto and PymoNNtorch depends greatly on the specific model at hand and the researcher's preferences. In situations where it is feasible to optimize various model operations, for example, through indexing techniques, the raw processing power of a GPU may not be necessary, and PymoNNto might be a suitable choice. On the other hand, for models where optimization opportunities are limited, and certain components of the model inherently benefit from GPU-accelerated computations, PymoNNtorch may be the preferred option. It is important to note that PymoNNtorch is still in its early stages of development and does not have the same set of high-level features, such as a GUI and evolution package, as PymoNNto has. We plan to add this high-level features by merging PymoNNto and PymoNNtorch into a common code base in the future while making sure that it does not affect the code of the described modules and measurements in this article. We invite the community to contribute to PymoNNto(rch)'s development.

We firmly believe that biologically plausible SNN models and a broader class of biologically inspired SNNs will continue to play a significant role in brain research and neuromorphic engineering, respectively. It is evident that the emergence of specialized hardware, such as neuromorphic chips, has the potential to provide even greater speed enhancements in SNN simulations. One example is Intel's previously developed chip called Loihi (Davies et al., 2018) and the corresponding Lava (Snyder et al., 2023) framework for implementing networks. However, we maintain the view that SNN simulations on standard hardware will remain essential for two reasons. First, standard hardware is cheap and widely available. Second, simulations on standard hardware can serve as valuable tools for assessing which features are worth integrating into the next generations of neuromorphic chips to enhance their capabilities.

## Data availability statement

The original contributions presented in the study are included in the article/Supplementary material, further inquiries can be directed to the corresponding authors.

## Author contributions

MV: Conceptualization, Data curation, Investigation, Methodology, Project administration, Software, Validation, Visualization, Writing – original draft, Writing – review & editing. AR: Conceptualization, Data curation, Investigation, Methodology, Project administration, Software, Validation, Visualization, Writing – original draft, Writing – review & editing. AG: Conceptualization, Investigation, Software, Writing – review & editing. JT: Project administration, Supervision, Writing – review & editing. MG: Project administration, Supervision, Writing – review & editing.

## Footnotes

^1^ https://pymonnto.readthedocs.io/

^2^ https://github.com/trieschlab/PymoNNto

^3^ https://github.com/cnrl/PymoNNtorch

^4^ https://pymonntorch.readthedocs.io/

^5^ https://github.com/saeedark/Fast-SNN-PymoNNto-rch
