# Supplementary material for: Accelerating spiking neural network simulations with PymoNNto and PymoNNtorch
Source: Front Neuroinform. 2024 Feb 20;18:1331220. doi: 10.3389/fninf.2024.1331220 (PMC10913591; doi:10.3389/fninf.2024.1331220)
Supplement: Supplementary file 1 [file Data_Sheet_1.pdf]

1 SUPPLEMENTARY MATERIALS

1.1 Sparse Implementation

To create a sparse connection in PymoNNto, an auxiliary backend like SciPy should be used. However, Pytorch, the backend of PymoNNtorch itself supports various sparse representations. Despite memory efficiency, sparse representations impose limitations on applicable operations. Yet a straightforward implementation of the LIF model with One-Step STDP is provided in the paper's repository on GitHub.

1.1.1 Synapse Operation

To perform the synapse function, the straightforward matrix-vector product can be employed:

```
"Numpy"
W.dot(src)

"PyTorch"
torch.matmul(W, src)
```

Here the W is a sparse matrix in **coo**rdinate representation. For a D×S matrix, this specific operation can be performed faster by using a Compressed Sparse Row(**CSR**) representation. However, the latter representation is not efficient for learning-related operations.

1.1.2 Synaptic Plasticity

The One-Step STDP discussed in the main text can be implemented as follows:

```
"Numpy"
mask = dst[W.row] * row[W.col]
W.data[mask] += 1

"PyTorch"
mask = dst[W.indices[0]] * row[W.indices[1]]
W._values()[mask] += 1
```

The $W.row$ and $W.indices[0]$ are the row coordinates of connections, indicating to which post-synaptic neuron the synapse is connected. Also, $W.col$ and $W.indices[1]$ refer to the column coordinates of connections, indicating to which pre-synaptic neuron the synapse is connected.

1.2 Simulator Setup

Each experiment run in this paper is executed independently from the others. The following details the setups for performing all the experiments:

1. The simulation resolution has been set to $1ms$ for all the simulators.
2. All simulators, except NEST (which only supports double precision) have been set to use single precision floating point.
3. NEST("local_num_threads") and ANNarchy("num_threads") are explicitly set to use 4 threads.

4. ANNarchy on CPU uses ”-O3” and ”-march=native” flags.
5. A fixed random seed is not set to ensure that our comparisons are valid and not influenced by one specific set of random numbers during all executions. Since NEST has a default seed, on each run a random seed has been set.

1.3 Model and Parameters: LIF Neurons with One-Step STDP

The model consists of a single neural population and an all-to-all synapse group connecting the neuron group to itself. Tables S1 and S2 represent the equations governing the population and synapse group, respectively. Table S3 lists the parameters and the values employed in this paper.

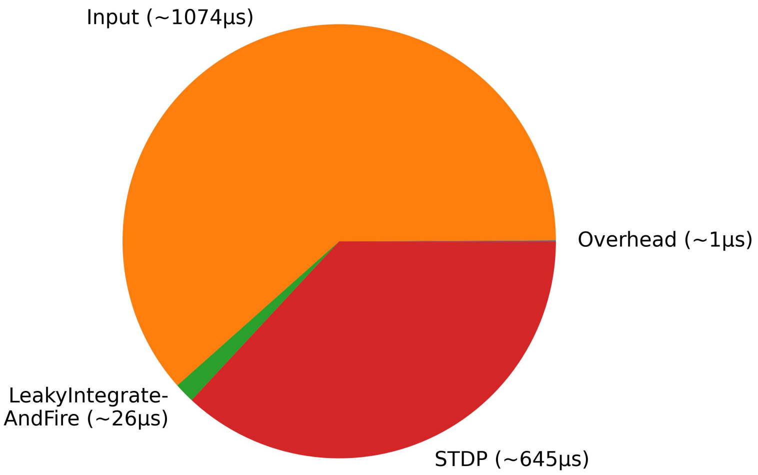

Figure S1: PymoNNto fast LIF with One-Step STDP model: Average share of computation time of one iteration of each module and the simulator overhead. Each module measurement is averaged over 300 iterations. To measure in the microsecond range, the simulator overhead was measured independently by computing the average over 100,000 iterations with “empty” iteration functions which only contain a simple “return True” command.

Neuron Model	
Type	Leaky integrate-and-fire
Subthreshold dynamics	$\tau \cdot \frac{dV}{dt} = -(V - V_{rest}) + I$
Spiking	$V > V_{threshold} \longrightarrow V = V_{rest}$

Table S1. The LIF neuronal model used in this experiment, where $I = I_{ext} + \sum_{syn} I_{syn}$ represents the external and synaptic currents coming into a neuron.

The LIF model with One-Step STDP implementation consists of the three Behavior Modules, namely “LeakyIntegrateAndFire”, “Input” and “STDP”. Here we show how the modules could be implemented

Synapse Model	
Type	Unit impulse, One-Step STDP
Dynamics	$I(t) = w \cdot \sum_{t_{pre}} \delta(t - t_{pre} - 1)$
Weight learning	$0 < (t_{post} - t_{pre}) \leq \Delta t \longrightarrow w = w + \eta$

Table S2. The synapse model with One-Step STDP. w, η, Δt, and δ represent synapse weight, learning rate, time resolution, and Dirac delta function respectively.

Parameters					
Name	$\tau(ms)$	$V_{init}(mV)$	$V_{rest}(mV)$	$V_{threshold}(mV)$	η
Value	10	0.0	0.0	6.0	0.001
Name	w	$I_{ext}(mV)$	Network size	Iterations	
Value	$\mathcal{U}_{[0,1]}$	$\mathcal{U}_{[0,1]}$	10,000	300	

Table S3. Parameters used with this model. $\mathcal{U}_{[a,b]}$ represents a uniform distribution form a to b. To easily scale the models, weights (w) are divided by the size of the pre-synaptic population.

in “PymoNNto (fast)”, “PymoNNto (naive)”, “PymoNNtorch (fast)” and “PymoNNtorch (naive)”. We also marked the NumPy and PyTorch code lines in yellow for which optimizations were discussed in the Methods section. If parts of the code blocks are annotated with “...”, it means that the missing code lines are the same as in the previous module.

```
######################################## PymoNNto (fast) ########################################
class LeakyIntegrateAndFire(Behavior):
    def initialize(self, neurons):
        neurons.spikes = neurons.vector('bool')
        neurons.spikesOld = neurons.vector('bool')
        neurons.voltage = neurons.vector()
        self.threshold = self.parameter('threshold')
        self.decay = self.parameter('decay')

    def iteration(self, neurons):
        neurons.spikesOld = neurons.spikes.copy()
        neurons.spikes = neurons.voltage > self.threshold
        neurons.voltage *= np.invert(neurons.spikes) #reset VR
        neurons.voltage *= self.decay #voltage decay

######################################## PymoNNto (naive) ########################################
class LeakyIntegrateAndFire(Behavior):
    def initialize(self, neurons): ...
    def iteration(self, neurons): ...

######################################## PymoNNtorch (fast) ########################################
class LeakyIntegrateAndFire(Behavior):

    def initialize(self, neurons):
        neurons.spikes = neurons.vector(dtype=torch.bool)
        neurons.spikesOld = neurons.vector(dtype=torch.bool)
        ...

    def forward(self, neurons):
        neurons.spikesOld = neurons.spikes.clone()
        ...
```

```
        neurons.voltage *= ~neurons.spikes #reset
        ...

####################################### PymoNNtorch (naive) #######################################
class LeakyIntegrateAndFire(Behavior):
    def initialize(self, neurons): ...
    def forward(self, neurons): ...
```

```
######################################### PymoNNto (fast) #########################################
class Input(Behavior):
    def initialize(self, neurons):
        for s in neurons.synapses(afferent, 'GLU'):
            s.W = s.matrix('random')
            s.W = s.W / SIZE

    def iteration(self, neurons):
        neurons.voltage += neurons.vector('random')
        for s in neurons.synapses(afferent, 'GLU'):
            s.dst.voltage += np.sum(s.W[s.src.spikes], axis=0)

######################################### PymoNNto (naive) #########################################
class Input(Behavior):
    def initialize(self, neurons): ...
    def iteration(self, neurons):
        ...
            s.dst.voltage += s.W.dot(s.src.spikes)
            ...

####################################### PymoNNtorch (fast) #######################################
class Input(Behavior):
    def initialize(self, neurons): ...
    def forward(self, neurons):
        ...
            s.dst.voltage += torch.sum(s.W[s.src.spikes], axis=0)
            ...

####################################### PymoNNtorch (naive) #######################################
class Input(Behavior):
    def initialize(self, neurons): ...
    def forward(self, neurons):
        ...
            s.dst.voltage += torch.tensordot(s.W, s.src.spikes.to(neurons.def_dtype), dims=[[1], [0]])
            ...
```

```
######################################### PymoNNto (fast) #########################################
class STDP(Behavior):
    def initialize(self, neurons):
        self.speed = self.parameter('speed')

    def iteration(self, neurons):
        for s in neurons.synapses(afferent, 'GLU'):
            mask = np.ix_(s.src.spikesOld, s.dst.spikes)
            s.W[mask] += self.speed
            s.W[mask] = np.clip(s.W[mask], 0.0, 1.0)

######################################### PymoNNto (naive) #########################################
class STDP(Behavior):
    def initialize(self, neurons): ...
```

```
    def iteration(self, neurons):
        ...
            s.W += s.dst.spikes[:, None] * s.src.spikesOld[None, :] * self.speed
            s.W = np.clip(s.W, 0.0, 1.0)

######################################## PymoNNtorch (fast) #######################################
class STDP(Behavior):
    def initialize(self, neurons): ...
    def forward(self, neurons):
        ...
            mask=(torch.where(s.src.spikesOld)[0].view(-1,1),torch.where(s.dst.spikes)[0].view(1,-1))
            s.W[mask] += self.speed
            s.W[mask] = torch.clip(s.W[mask], 0.0, 1.0)

####################################### PymoNNtorch (naive) #######################################
class STDP(Behavior):
    def initialize(self, neurons): ...

    def forward(self, neurons):
        ...
            s.W += s.dst.spikes[:, None] * s.src.spikesOld[None, :] * self.speed
            s.W = torch.clip(s.W, 0.0, 1.0)
```

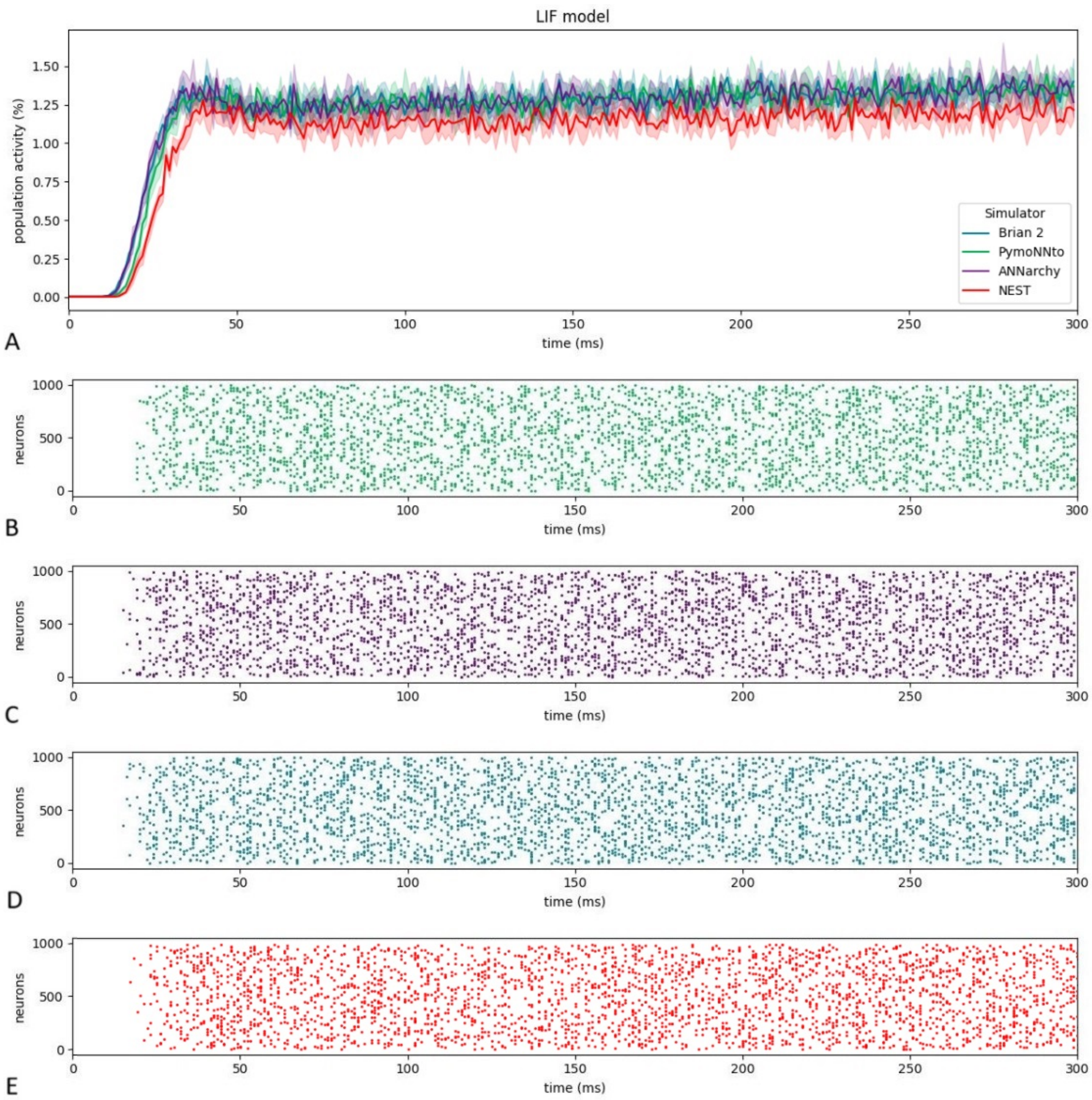

Figure S2: A: Mean population activity (share of neurons which are active during 1ms, averaged over five runs) of the LIF with One-Step STDP model for PymoNNto (green), Brian 2 (blue), ANNarchy (purple), and NEST (red). B-E: Sample spike activity (first 1,000 out of 10,000 neurons) for each of the four simulators in the same coloration as above (B: PymoNNto, C: Annarchy, D: Brian, E: NEST). We only show the CPU variants of each simulator and did not show PymoNNtorch, because the output of its operations is equivalent to PymoNNto. Note that NEST (red) has a slightly lower firing rate despite using the same equations and parameters (This is not caused by its double precision). All the other simulators generate the same population activity.

1.4 Model and Parameters: Izhikevich Neurons with Standard STDP

This model also consists of a single neural population and an all-to-all synapse group connecting the neuron group to itself. Tables S4 and S5 represent the equations governing the population and synapse, respectively. Table S6 lists the parameters and the values employed in this paper.

It is important to note that our model employs a step size of 1ms for the sake of simplicity, a choice that has not posed issues within the context of our model. Nevertheless, it is crucial to acknowledge that using such a large step size can lead to distortions in the dynamics of Izhikevich neurons with specific sets of parameters, as highlighted in Pauli et al. (2018).

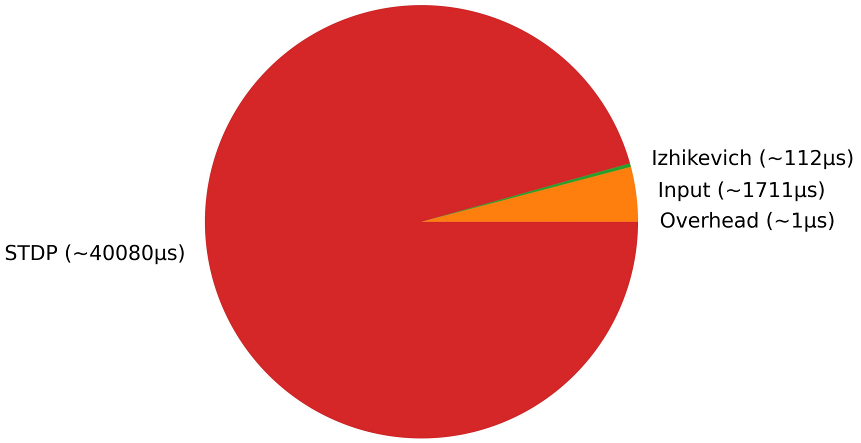

Figure S3: PymoNNto Izhikevich with standard STDP model: Average share of computation time in one iteration of each module and the simulator overhead. Each module measurement is averaged over 300 iterations. To measure in the microsecond range, the simulator overhead was measured independently by computing the average over 100,000 iterations with "empty" iteration functions which only contain a simple "return True" command.

Neuron Model	
Type	The Izhikevich neuronal model
Subthreshold dynamics	$\frac{dV}{dt} = 0.04V^2 + 5V + 140 - u + I$ $\frac{dU}{dt} = a(bV - U)$
Spiking	$V \geq 30.0 \longrightarrow V = c\,, U = U + d$

Table S4. The original Izhikevich neuronal model. Just like the previous model, $I = I_{ext} + \sum_{syn} I_{syn}$ represents the external and synaptic currents coming into a neuron.

Synapse Model	
Type	Unit impulse, Standard STDP with Multiplicative Weight Dependence
Dynamics	$I(t) = w \cdot \sum_{t_{pre}} \delta(t - t_{pre} - 1)$
Weight learning	$\frac{dx}{dt} = -\frac{x}{tau_-} + \sum_{t_{pre}} \delta(t - t_{pre})$ $\frac{dy}{dt} = -\frac{y}{tau_+} + \sum_{t_{post}} \delta(t - t_{post})$ $\Delta w = -(\eta_- \cdot x \cdot (w - w_{min}) \cdot \sum_{t_{pre}} \delta(t - t_{pre}))$ $+(\eta_+ \cdot y \cdot (w_{max} - w) \cdot \sum_{t_{post}} \delta(t - t_{post}))$

Table S5. The synapse model with the standard STDP. x and y represent the spike trace of pre and post-synaptic neurons respectively.

Parameters								
Name	a	b	$c(mV)$	$d(mV)$	$\tau_+(ms)$	$\tau_-(ms)$	w_{min}	w_{max}
Value	0.02	0.04	-65	2	20	20	0.0	1.0
Name	η_+	η_-	$V_{init}(mV)$	$U_{init}(mV)$	w_{init}	$I_{ext}(mV)$	Network size	Iterations
Value	0.01	0.012	$\mathcal{N}(-65, 7)$	$\mathcal{N}(12, 7)$	$\mathcal{U}_{[0,1]}$	$\mathcal{N}(0, 1)$	10,000	300

Table S6. Model parameters. $\mathcal{N}(a, b)$ represents a normal distribution with mean a and standard deviation b. To scale models easily, the effect of weights (w) is divided by pre-synaptic population size.

The Izhikevich Standard STDP implementation consists of the three Behavior Modules, namely "Izhikevich", "Input" and "STDP". Here we show how these modules could be implemented in different versions of PymoNNto(rch). We also marked the NumPy and PyTorch code lines in yellow for which optimizations were discussed in the Methods section. If parts of the code blocks are annotated with "..." it means that the missing code lines are the same as in the previous module.

```
############################################ PymoNNto ############################################
class Izhikevich(Behavior):
    def initialize(self, n):
        self.a = self.parameter("a")
        self.b = self.parameter("b")
        self.c = self.parameter("c")
        self.d = self.parameter("d")
        self.threshold = self.parameter("threshold")

        n.v = V_STD * n.vector("normal") + V_MEAN
        n.u = U_STD * n.vector("normal") + U_MEAN
        n.spikes = n.vector("bool")

    def iteration(self, n):
        n.spikes = (n.v >= self.threshold)

        n.v[n.spikes] = self.c
        n.u[n.spikes] += self.d

        dv = (0.04 * n.v**2.0 + 5.0 * n.v + 140.0 - n.u + n.I)
        du = (self.a * (self.b * n.v - n.u))

        n.v += dv * n.network.dt
        n.u += du * n.network.dt
```

```python
############################################ PymoNNtorch ############################################
class Izhikevich(Behavior):
    def initialize(self, n):
        ...
        n.u = n.vector(f"normal({U_MEAN}, {U_STD})")
        n.v = n.vector(f"normal({V_MEAN}, {V_STD})")
        n.spikes = n.vector(dtype=torch.bool)

    def forward(self, n): ...
```

```python
############################################ PymoNNto ############################################
class Input(Behavior):
    def initialize(self, n):
        self.offset = self.parameter("offset")
        self.strength = self.parameter("strength")
        n.I = n.vector(self.offset)
        for s in n.afferent_synapses["GLU"]:
            s.W = s.matrix("random") * W_MAX + W_MIN

    def iteration(self, n):
        n.I.fill(self.offset)
        n.I += n.vector("normal") * NOISE_STD + NOISE_MEAN

        for s in n.afferent_synapses["GLU"]:
            n.I += np.sum(s.W[s.src.spikes], axis=0) * self.strength

############################################ PymoNNtorch ############################################
class Input(Behavior):
    def initialize(self, n):
        self.offset = self.parameter("offset")
        self.strength = self.parameter("strength")
        n.I = n.vector(self.offset)
        for s in n.afferent_synapses["GLU"]:
            s.W = s.matrix("random") * W_MAX + W_MIN

    def forward(self, n):
        n.I.fill_(self.offset)
        for s in n.afferent_synapses["GLU"]:
            n.I += torch.sum(s.W[s.src.spikes], axis=0) * self.strength
        n.I += n.vector(f"normal({NOISE_MEAN}, {NOISE_STD})")
```

```python
############################################ PymoNNto ############################################
class STDP(Behavior):
    def initialize(self, s):
        self.pre_tau = self.parameter("pre_tau")
        self.post_tau = self.parameter("post_tau")
        self.a_plus = self.parameter("a_plus")
        self.a_minus = self.parameter("a_minus")

        s.src_trace = s.src.vector()
        s.dst_trace = s.dst.vector()

    def iteration(self, s):
        src_spikes = s.src.spikes
        dst_spikes = s.dst.spikes
        s.src_trace += src_spikes - s.src_trace / self.pre_tau * s.network.dt
        s.dst_trace += dst_spikes - s.dst_trace / self.post_tau * s.network.dt
        s.W[src_spikes] -= (
```

```
            s.dst_trace[None, ...] * self.a_minus * (s.W[src_spikes] - W_MIN)
        )
        s.W[:, dst_spikes] += (
            s.src_trace[..., None] * self.a_plus * (W_MAX - s.W[:, dst_spikes])
        )

########################################### PymoNNtorch ###########################################
class STDP(Behavior):
    def initialize(self, s): ...
    def forward(self, s):
        ...
        s.src_trace += src_spikes * 1.0 - s.src_trace / self.pre_tau * s.network.dt
        s.dst_trace += dst_spikes * 1.0 - s.dst_trace / self.post_tau * s.network.dt
        ...
```

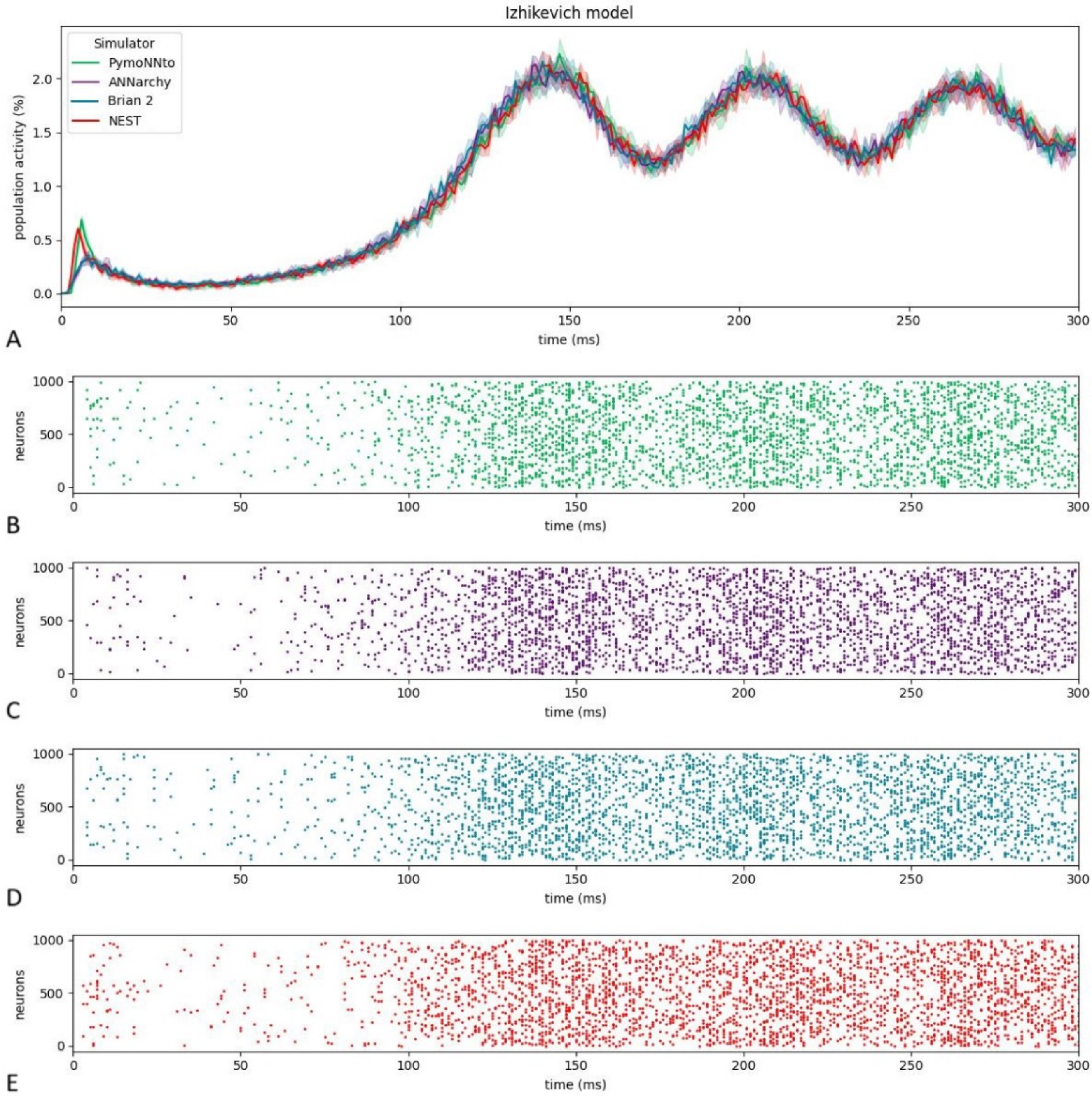

Figure S4: A: Mean population activity (share of neurons which are active during 1ms, averaged over five runs) of the Izhikevich with standard STDP model for PymoNNto (green), Brian 2 (blue), ANNarchy (purple) and NEST (red). B-E: Sample spike activity (first 1,000 out of 10,000 neurons) for each of the four simulators in the same coloration as above (B: PymoNNto, C: Annarchy, D: Brian, E: NEST). We only show the CPU variants of each simulator and did not show PymoNNtorch, because the output of its operations is equivalent to PymoNNto. The simulators generate the same population activity.

1.5 Simulators and Device Specification

All measurements were conducted on the same device with the same software setup as described in Table S7.

Hard- and Software	Version/Name
Device	Amazon AWS EC2 g4dn.xlarge
CPU	Intel® Xeon Cascade Lake P-8259CL 4x vCPU
GPU	Nvidia T4 Tensor Core
OS Base Image	Deep Learning AMI GPU PyTorch 2.1.0 (Ubuntu 20.04) 20231103
Python	3.10.9
NumPy	1.26.2
PyTorch	2.1.1
CUDA	12.2
nvcc	V12.1.105
Nvidia driver	535.104.12
Brian2	2.5.4
Brian2Cuda	1.0a3
ANNarchy	4.7.2.6
PyNN	0.12.1
NEST	3.5
NESTML	5.3.0.post0.dev0
PymoNNto	3.0.1
PymoNNtorch	0.1.3

Table S7. Hardware and software versions utilized to conduct the experiments.

1.6 Network Scaling and Firing Rates

Here we compare different population sizes and three parameters affecting the firing rate. Figures S5 and S6 show how the firing rates and the simulation times change under these conditions. As mentioned in the text, the weights of synapses are scaled by the size of the pre-synaptic population in order to have similar firing rates. For the LIF model, we changed $V_{threshold}$ to 5.6 (high firing rate), 6.0 (medium firing rate), or 6.7 (low firing rate). For the Izhikevich model, we modified I_{ext} to $\mathcal{N}(-1.55, 1)$(low firing rate), $\mathcal{N}(0, 1)$(medium firing rate) and $\mathcal{N}(1.3, 1)$(high firing rate). Figure S6 indicates that as the spike rate increases, PymoNNto(rch) can be faster on smaller networks.

1.7 Simulation time

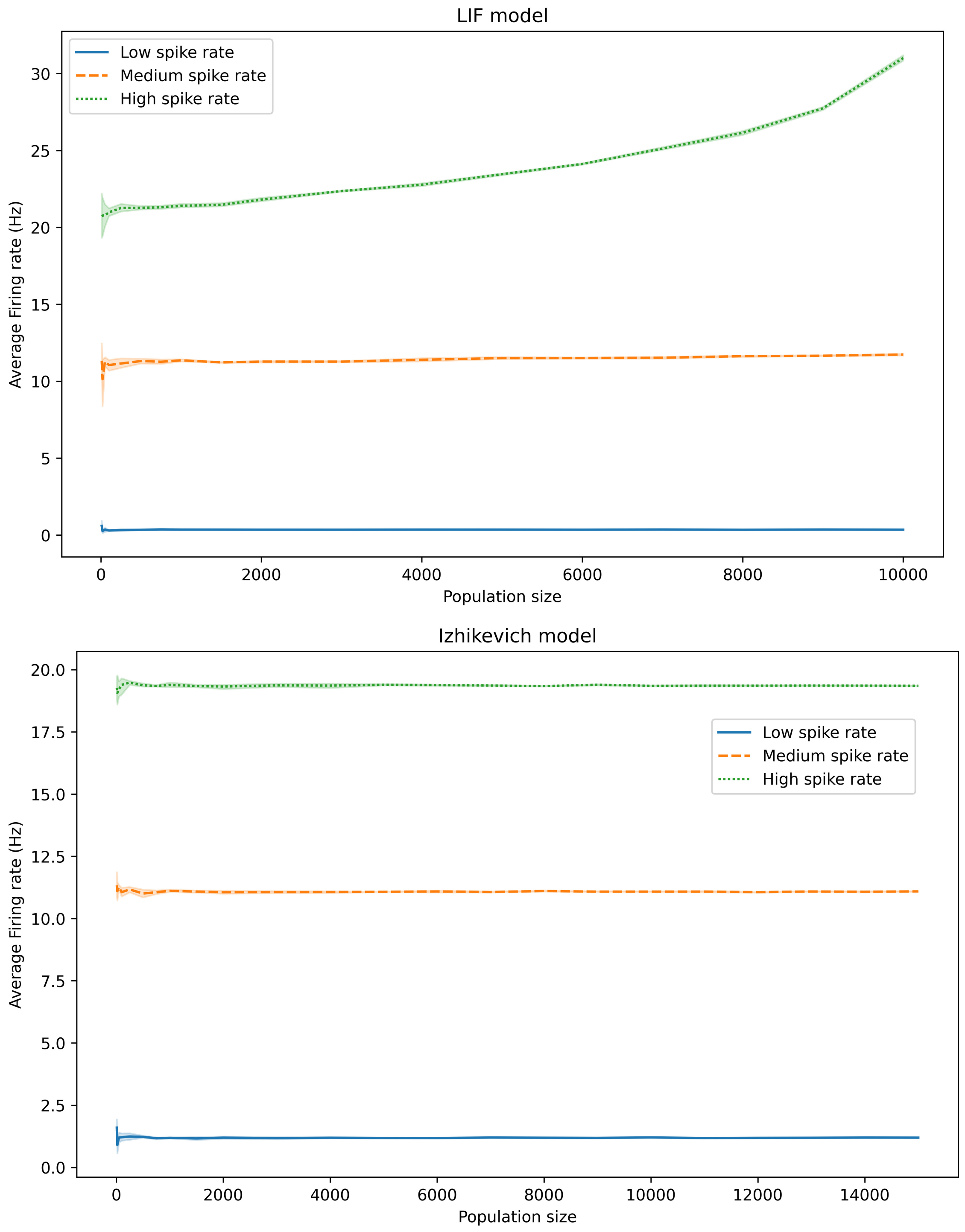

Figure S5: Top: The LIF model and firing rates for three different parameters in comparison to the increase of population size. Note that even though weights are normalized by the size of the neuronal population, the learning rate is constant in all three, thus there is an increase in firing rate as population size grows. Bottom: The Izhikevich model and firing rates for three different parameters in comparison to the increase in population size.

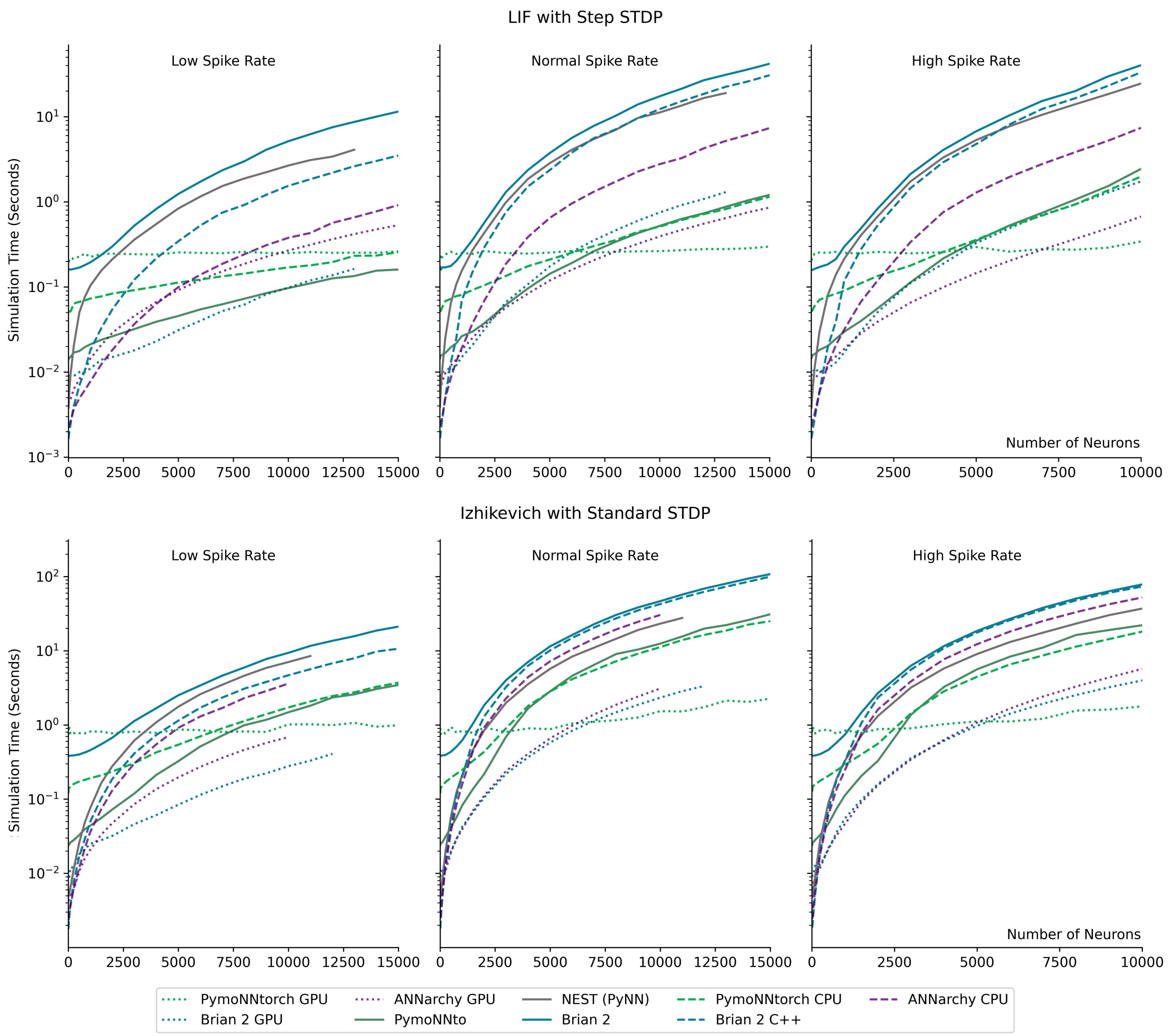

Figure S6: The LIF model (top) and the Izhikevich model (bottom) with three different firing rates (low, medium and high) for different population sizes.

One-Step STDP	*naive*				*efficient*			
Synapse Operation	*naive*		*efficient*		*naive*		*efficient*	
Data Type	$float64$	$float32$	$float62$	$float32$	$float64$	$float32$	$float64$	$float32$
PymoNNto	295.31 ± 0.665	239.588 ± 0.951	262.543 ± 0.614	222.465 ± 0.518	9.73 ± 0.03	5.062 ± 0.03	0.764 ± 0.014	0.534 ± 0.007
PymoNNtorch CPU	330.035 ± 0.889	177.286 ± 0.236	314.083 ± 0.66	173.062 ± 0.492	9.69 ± 0.019	4.776 ± 0.008	0.693 ± 0.027	0.503 ± 0.006
PymoNNtorch GPU	5.628 ± 0.028	3.052 ± 0.003	5.855 ± 0.01	3.57 ± 0.007	1.708 ± 0.013	0.739 ± 0.01	0.289 ± 0.012	0.281 ± 0.015

Table S8. Simulation times (milliseconds) of various implementations of the LIF model in PymoNNto(rch) (averaged over five repetitions). Data type row indicates if the simulation uses single precision ($float32$) instead of double precision ($float64$). Synapse Operation and One-Step STDP rows indicate if the simulation uses the corresponding efficient implementation instead of the naive one.

Network size	Simulators								
	ANNarchy CPU	ANNarchy GPU	Brian 2	Brian 2 C++	Brian 2 GPU	NEST	PymoNNto	PymoNNtorch CPU	PymoNNtorch GPU
10	0.002 (90.333×)	0.004 (48.461×)	0.16 (1.273×)	**0.002 (124.405×)**	0.009 (22.683×)	0.004 (53.184×)	0.014 (14.377×)	0.051 (3.992×)	0.204 (1.0×)
20	0.002 (80.076×)	0.004 (42.987×)	0.162 (1.149×)	**0.002 (105.838×)**	0.009 (20.697×)	0.004 (42.057×)	0.015 (12.802×)	0.051 (3.658×)	0.186 (1.0×)
50	0.002 (86.819×)	0.005 (45.6×)	0.16 (1.309×)	**0.002 (101.59×)**	0.009 (23.275×)	0.006 (32.789×)	0.015 (14.278×)	0.051 (4.074×)	0.209 (1.0×)
100	0.003 (77.541×)	0.005 (42.48×)	0.16 (1.284×)	**0.002 (82.749×)**	0.009 (22.793×)	0.009 (21.731×)	0.015 (13.6×)	0.051 (3.984×)	0.205 (1.0×)
250	**0.004 (59.655×)**	0.006 (34.724×)	0.163 (1.35×)	0.004 (54.732×)	0.009 (24.435×)	0.021 (10.648×)	0.017 (12.976×)	0.064 (3.445×)	0.22 (1.0×)
500	**0.005 (45.841×)**	0.008 (27.382×)	0.169 (1.339×)	0.007 (32.607×)	0.01 (22.596×)	0.05 (4.492×)	0.018 (12.795×)	0.067 (3.379×)	0.226 (1.0×)
750	**0.006 (40.199×)**	0.01 (23.518×)	0.181 (1.365×)	0.01 (23.529×)	0.01 (24.677×)	0.076 (3.233×)	0.02 (12.552×)	0.069 (3.557×)	0.247 (1.0×)
1000	**0.008 (28.977×)**	0.014 (15.688×)	0.194 (1.157×)	0.018 (12.454×)	0.011 (20.458×)	0.104 (2.171×)	0.021 (10.629×)	0.073 (3.081×)	0.225 (1.0×)
1500	**0.012 (19.785×)**	0.021 (11.727×)	0.237 (1.029×)	0.033 (7.427×)	0.014 (17.45×)	0.156 (1.565×)	0.024 (10.194×)	0.077 (3.153×)	0.244 (1.0×)
2000	0.018 (16.46×)	0.029 (10.339×)	0.299 (1.0×)	0.055 (5.48×)	**0.015 (19.935×)**	0.212 (1.413×)	0.026 (11.379×)	0.084 (3.577×)	0.246 (1.215×)
3000	0.037 (14.306×)	0.046 (11.467×)	0.525 (1.0×)	0.122 (4.308×)	**0.018 (29.166×)**	0.359 (1.461×)	0.032 (16.409×)	0.092 (5.721×)	0.243 (2.164×)
4000	0.063 (13.059×)	0.067 (12.406×)	0.827 (1.0×)	0.217 (3.806×)	**0.023 (35.937×)**	0.549 (1.506×)	0.039 (21.195×)	0.101 (8.164×)	0.24 (3.442×)
5000	0.099 (12.447×)	0.092 (13.463×)	1.237 (1.0×)	0.344 (3.596×)	**0.031 (39.908×)**	0.834 (1.483×)	0.046 (27.138×)	0.113 (10.973×)	0.255 (4.856×)
6000	0.139 (12.393×)	0.12 (14.297×)	1.722 (1.0×)	0.526 (3.271×)	**0.04 (43.051×)**	1.151 (1.496×)	0.054 (31.638×)	0.122 (14.11×)	0.251 (6.857×)
7000	0.187 (12.57×)	0.153 (15.376×)	2.346 (1.0×)	0.746 (3.143×)	**0.052 (45.118×)**	1.531 (1.532×)	0.063 (37.497×)	0.133 (17.601×)	0.249 (9.424×)
8000	0.242 (12.382×)	0.187 (16.032×)	2.993 (1.0×)	0.919 (3.255×)	**0.062 (48.271×)**	1.878 (1.593×)	0.073 (41.196×)	0.143 (20.864×)	0.259 (11.563×)
9000	0.309 (13.234×)	0.226 (18.112×)	4.089 (1.0×)	1.209 (3.382×)	**0.082 (49.869×)**	2.228 (1.835×)	0.085 (48.385×)	0.157 (26.117×)	0.251 (16.3×)
10000	0.377 (13.633×)	0.268 (19.203×)	5.144 (1.0×)	1.539 (3.343×)	0.098 (52.49×)	2.666 (1.929×)	**0.097 (53.044×)**	0.17 (30.333×)	0.25 (20.587×)
11000	0.43 (14.448×)	0.312 (19.901×)	6.219 (1.0×)	1.84 (3.379×)	0.119 (52.258×)	3.09 (2.012×)	**0.11 (56.536×)**	0.179 (34.665×)	0.257 (24.187×)
12000	0.565 (13.281×)	0.363 (20.661×)	7.506 (1.0×)	2.193 (3.423×)	0.137 (54.788×)	3.391 (2.213×)	**0.126 (59.581×)**	0.195 (38.459×)	0.251 (29.876×)
13000	0.657 (13.208×)	0.418 (20.776×)	8.676 (1.0×)	2.618 (3.314×)	0.163 (53.229×)	4.092 (2.12×)	**0.134 (64.669×)**	0.232 (37.398×)	0.253 (34.356×)
14000	0.771 (12.982×)	0.474 (21.114×)	10.008 (1.0×)	3.012 (3.323×)	—	—	**0.155 (64.494×)**	0.233 (42.916×)	0.252 (39.718×)
15000	0.914 (12.555×)	0.531 (21.603×)	11.476 (1.0×)	3.493 (3.285×)	—	—	**0.16 (71.659×)**	0.257 (44.69×)	0.261 (43.891×)

Table S9. Simulation times (seconds) of different simulations for the network of **low-firing LIF** neurons with respect to the network size (single run). The ratio of how much faster the simulator is over the slowest one with the same network size is shown in parentheses. The fastest simulator with respect to the network size is highlighted in bold. The empty cells represent instances where the system ran out of memory.

Network size	Simulators								
	ANNarchy CPU	ANNarchy GPU	Brian 2	Brian 2 C++	Brian 2 GPU	NEST	PymoNNto	PymoNNtorch CPU	PymoNNtorch GPU
10	0.002 (97.591×)	0.005 (50.554×)	0.172 (1.344×)	**0.002 (137.978×)**	0.009 (25.649×)	0.004 (59.066×)	0.015 (15.914×)	0.051 (4.546×)	0.231 (1.0×)
20	0.002 (86.531×)	0.005 (41.879×)	0.161 (1.302×)	**0.002 (119.437×)**	0.009 (23.304×)	0.005 (46.122×)	0.015 (14.051×)	0.051 (4.076×)	0.21 (1.0×)
50	0.003 (92.779×)	0.007 (35.604×)	0.159 (1.507×)	**0.002 (115.615×)**	0.01 (24.025×)	0.007 (36.82×)	0.016 (15.291×)	0.053 (4.572×)	0.24 (1.0×)
100	0.003 (78.48×)	0.008 (29.405×)	0.169 (1.313×)	**0.003 (83.868×)**	0.01 (22.225×)	0.01 (22.211×)	0.016 (13.989×)	0.057 (3.888×)	0.222 (1.0×)
250	**0.005 (49.0×)**	0.01 (24.969×)	0.17 (1.417×)	0.005 (47.615×)	0.01 (24.074×)	0.025 (9.578×)	0.017 (14.363×)	0.068 (3.536×)	0.241 (1.0×)
500	**0.009 (30.367×)**	0.012 (21.714×)	0.176 (1.471×)	0.013 (20.175×)	0.01 (25.899×)	0.064 (4.016×)	0.02 (13.28×)	0.072 (3.577×)	0.259 (1.0×)
750	0.013 (18.217×)	0.014 (17.426×)	0.202 (1.21×)	0.025 (9.808×)	**0.012 (20.363×)**	0.107 (2.276×)	0.022 (11.231×)	0.078 (3.131×)	0.244 (1.0×)
1000	0.019 (13.14×)	0.018 (13.673×)	0.245 (1.026×)	0.069 (3.614×)	**0.015 (16.733×)**	0.155 (1.62×)	0.026 (9.507×)	0.081 (3.113×)	0.251 (1.0×)
1500	0.038 (9.522×)	0.025 (14.238×)	0.361 (1.0×)	0.152 (2.37×)	**0.021 (17.168×)**	0.274 (1.314×)	0.03 (12.015×)	0.092 (3.9×)	0.244 (1.479×)
2000	0.067 (8.286×)	0.035 (15.889×)	0.557 (1.0×)	0.285 (1.956×)	**0.031 (17.976×)**	0.424 (1.315×)	0.037 (15.046×)	0.104 (5.366×)	0.264 (2.109×)
3000	0.186 (6.995×)	**0.057 (22.714×)**	1.299 (1.0×)	0.751 (1.728×)	0.066 (19.677×)	0.982 (1.322×)	0.061 (21.136×)	0.135 (9.654×)	0.25 (5.195×)
4000	0.384 (6.125×)	**0.083 (28.283×)**	2.353 (1.0×)	1.507 (1.562×)	0.107 (21.989×)	1.848 (1.273×)	0.096 (24.592×)	0.175 (13.447×)	0.247 (9.541×)
5000	0.647 (5.777×)	**0.119 (31.46×)**	3.737 (1.0×)	2.355 (1.587×)	0.174 (21.477×)	2.843 (1.315×)	0.143 (26.219×)	0.21 (17.817×)	0.253 (14.782×)
6000	0.954 (5.894×)	**0.159 (35.365×)**	5.624 (1.0×)	3.78 (1.488×)	0.258 (21.799×)	4.082 (1.378×)	0.191 (29.45×)	0.252 (22.275×)	0.266 (21.172×)
7000	1.313 (5.931×)	**0.206 (37.845×)**	7.786 (1.0×)	5.584 (1.394×)	0.352 (22.119×)	5.45 (1.429×)	0.262 (29.707×)	0.302 (25.799×)	0.253 (30.788×)
8000	1.725 (5.944×)	0.261 (39.277×)	10.252 (1.0×)	7.102 (1.443×)	0.462 (22.19×)	7.003 (1.464×)	0.339 (30.231×)	0.355 (28.895×)	**0.259 (39.589×)**
9000	2.259 (6.158×)	0.321 (43.389×)	13.91 (1.0×)	9.585 (1.451×)	0.598 (23.261×)	9.634 (1.444×)	0.428 (32.488×)	0.444 (31.364×)	**0.262 (53.111×)**
10000	2.768 (6.273×)	0.392 (44.272×)	17.363 (1.0×)	12.265 (1.416×)	0.749 (23.182×)	11.207 (1.549×)	0.521 (33.3×)	0.511 (33.957×)	**0.264 (65.877×)**
11000	3.263 (6.537×)	0.468 (45.548×)	21.327 (1.0×)	15.2 (1.403×)	0.917 (23.257×)	13.532 (1.576×)	0.63 (33.856×)	0.61 (34.957×)	**0.27 (78.88×)**
12000	4.244 (6.314×)	0.552 (48.508×)	26.792 (1.0×)	18.503 (1.448×)	1.079 (24.831×)	16.572 (1.617×)	0.732 (36.591×)	0.716 (37.419×)	**0.278 (96.251×)**
13000	5.183 (5.989×)	0.643 (48.257×)	31.038 (1.0×)	22.446 (1.383×)	1.299 (23.894×)	18.935 (1.639×)	0.873 (35.548×)	0.82 (37.84×)	**0.278 (111.448×)**
14000	6.122 (5.855×)	0.751 (47.754×)	35.842 (1.0×)	25.894 (1.384×)	—	—	1.038 (34.515×)	0.979 (36.613×)	**0.284 (126.266×)**
15000	7.36 (5.692×)	0.857 (48.894×)	41.896 (1.0×)	30.691 (1.365×)	—	—	1.207 (34.709×)	1.145 (36.583×)	**0.298 (140.562×)**

Table S10. Simulation times (seconds) of different simulations for the network of **medium-firing LIF** neurons with respect to the network size (single run). The ratio of how much faster the simulator is over the slowest one with the same network size is shown in parentheses. The fastest simulator with respect to the network size is highlighted in bold. The empty cells represent instances where the system ran out of memory.

Network size	Simulators								
	ANNarchy CPU	ANNarchy GPU	Brian 2	Brian 2 C++	Brian 2 GPU	NEST	PymoNNto	PymoNNtorch CPU	PymoNNtorch GPU
10	0.002 (89.433×)	0.005 (40.931×)	0.158 (1.3×)	**0.002 (123.887×)**	0.009 (22.836×)	0.004 (53.908×)	0.015 (14.048×)	0.051 (4.03×)	0.206 (1.0×)
20	0.002 (86.141×)	0.005 (38.651×)	0.159 (1.327×)	**0.002 (118.926×)**	0.01 (21.133×)	0.005 (46.608×)	0.015 (14.118×)	0.052 (4.056×)	0.211 (1.0×)
50	0.003 (80.472×)	0.008 (27.85×)	0.16 (1.374×)	**0.002 (103.039×)**	0.01 (21.916×)	0.007 (32.491×)	0.016 (13.746×)	0.054 (4.036×)	0.219 (1.0×)
100	0.003 (77.63×)	0.008 (29.551×)	0.163 (1.514×)	**0.003 (88.681×)**	0.011 (22.412×)	0.011 (22.541×)	0.016 (15.228×)	0.06 (4.139×)	0.247 (1.0×)
250	**0.006 (43.876×)**	0.01 (26.084×)	0.171 (1.476×)	0.006 (42.902×)	0.01 (25.312×)	0.03 (8.533×)	0.018 (13.906×)	0.071 (3.588×)	0.253 (1.0×)
500	0.012 (20.81×)	0.013 (19.958×)	0.184 (1.365×)	0.019 (12.924×)	**0.011 (22.83×)**	0.08 (3.147×)	0.02 (12.395×)	0.077 (3.245×)	0.251 (1.0×)
750	0.021 (12.444×)	0.015 (16.924×)	0.213 (1.215×)	0.04 (6.527×)	**0.013 (19.888×)**	0.14 (1.844×)	0.025 (10.496×)	0.083 (3.106×)	0.259 (1.0×)
1000	0.031 (9.422×)	0.019 (15.88×)	0.297 (1.0×)	0.118 (2.525×)	**0.017 (17.457×)**	0.214 (1.39×)	0.03 (9.966×)	0.09 (3.283×)	0.251 (1.182×)
1500	0.066 (7.28×)	**0.028 (16.986×)**	0.483 (1.0×)	0.274 (1.762×)	0.03 (16.114×)	0.401 (1.205×)	0.04 (12.201×)	0.11 (4.381×)	0.248 (1.953×)
2000	0.117 (7.009×)	**0.039 (21.231×)**	0.821 (1.0×)	0.52 (1.578×)	0.05 (16.415×)	0.664 (1.236×)	0.055 (14.794×)	0.134 (6.13×)	0.258 (3.177×)
3000	0.333 (6.32×)	**0.065 (32.32×)**	2.106 (1.0×)	1.436 (1.467×)	0.111 (18.977×)	1.722 (1.223×)	0.113 (18.715×)	0.178 (11.849×)	0.248 (8.5×)
4000	0.754 (5.385×)	**0.099 (40.975×)**	4.061 (1.0×)	2.914 (1.394×)	0.187 (21.717×)	3.314 (1.225×)	0.216 (18.811×)	0.258 (15.721×)	0.256 (15.845×)
5000	1.284 (5.243×)	**0.146 (46.231×)**	6.73 (1.0×)	4.769 (1.411×)	0.327 (20.58×)	5.318 (1.265×)	0.348 (19.348×)	0.357 (18.838×)	0.294 (22.869×)
6000	1.95 (5.29×)	**0.202 (51.096×)**	10.316 (1.0×)	8.073 (1.278×)	0.488 (21.138×)	7.674 (1.344×)	0.526 (19.629×)	0.513 (20.116×)	0.26 (39.671×)
7000	2.778 (5.506×)	**0.274 (55.836×)**	15.294 (1.0×)	12.323 (1.241×)	0.702 (21.786×)	10.526 (1.453×)	0.742 (20.606×)	0.69 (22.16×)	0.278 (54.973×)
8000	3.827 (5.214×)	0.365 (54.627×)	19.957 (1.0×)	16.452 (1.213×)	0.933 (21.39×)	13.981 (1.427×)	1.061 (18.81×)	0.93 (21.457×)	**0.274 (72.853×)**
9000	5.22 (5.693×)	0.489 (60.748×)	29.721 (1.0×)	23.048 (1.29×)	1.287 (23.093×)	18.412 (1.614×)	1.529 (19.441×)	1.365 (21.775×)	**0.289 (102.764×)**
10000	7.4 (5.436×)	0.67 (60.078×)	40.228 (1.0×)	33.244 (1.21×)	1.734 (23.199×)	24.539 (1.639×)	2.436 (16.511×)	1.978 (20.335×)	**0.342 (117.578×)**

Table S11. Simulation times (seconds) of different simulations for the network of **high-firing LIF** neurons with respect to the network size (single run). The ratio of how much faster the simulator is over the slowest one with the same network size is shown in parentheses. The fastest simulator with respect to the network size is highlighted in bold.

Network size	Simulators								
	ANNarchy CPU	ANNarchy GPU	Brian 2	Brian 2 C++	Brian 2 GPU	NEST	PymoNNto	PymoNNtorch CPU	PymoNNtorch GPU
10	0.002 (392.044×)	0.004 (214.67×)	0.384 (2.376×)	**0.002 (512.906×)**	0.009 (101.384×)	0.006 (152.209×)	0.024 (38.292×)	0.121 (7.541×)	0.912 (1.0×)
20	0.002 (314.284×)	0.004 (174.898×)	0.384 (1.975×)	**0.002 (377.118×)**	0.009 (84.181×)	0.005 (154.139×)	0.025 (30.895×)	0.144 (5.25×)	0.758 (1.0×)
50	0.003 (304.215×)	0.005 (194.676×)	0.384 (2.384×)	**0.003 (340.464×)**	0.009 (101.647×)	0.005 (168.536×)	0.025 (37.037×)	0.143 (6.381×)	0.915 (1.0×)
100	**0.004 (205.815×)**	0.005 (142.1×)	0.386 (1.93×)	0.004 (197.203×)	0.012 (62.07×)	0.006 (115.663×)	0.026 (28.592×)	0.145 (5.15×)	0.745 (1.0×)
250	0.006 (123.843×)	**0.006 (128.527×)**	0.388 (1.997×)	0.008 (100.837×)	0.012 (64.611×)	0.011 (67.623×)	0.028 (27.593×)	0.16 (4.837×)	0.775 (1.0×)
500	0.012 (65.664×)	**0.011 (72.495×)**	0.401 (1.933×)	0.017 (46.088×)	0.018 (43.031×)	0.026 (29.904×)	0.033 (23.634×)	0.173 (4.48×)	0.775 (1.0×)
750	0.022 (35.506×)	**0.016 (48.626×)**	0.425 (1.81×)	0.029 (26.64×)	0.021 (36.614×)	0.048 (15.885×)	0.039 (19.578×)	0.182 (4.225×)	0.769 (1.0×)
1000	0.036 (22.653×)	**0.021 (39.273×)**	0.456 (1.79×)	0.05 (16.392×)	0.025 (32.659×)	0.076 (10.764×)	0.044 (18.605×)	0.192 (4.262×)	0.816 (1.0×)
1500	0.073 (11.073×)	0.033 (24.422×)	0.549 (1.48×)	0.102 (7.939×)	**0.028 (29.011×)**	0.165 (4.925×)	0.056 (14.588×)	0.209 (3.877×)	0.812 (1.0×)
2000	0.131 (5.886×)	0.047 (16.251×)	0.673 (1.145×)	0.187 (4.123×)	**0.032 (24.094×)**	0.278 (2.777×)	0.073 (10.633×)	0.236 (3.271×)	0.771 (1.0×)
3000	0.304 (3.714×)	0.085 (13.2×)	1.128 (1.0×)	0.412 (2.74×)	**0.046 (24.525×)**	0.618 (1.826×)	0.119 (9.494×)	0.306 (3.691×)	0.814 (1.385×)
4000	0.547 (3.078×)	0.137 (12.325×)	1.683 (1.0×)	0.732 (2.299×)	**0.061 (27.595×)**	1.087 (1.548×)	0.212 (7.929×)	0.427 (3.94×)	0.782 (2.151×)
5000	0.914 (2.746×)	0.196 (12.79×)	2.51 (1.0×)	1.142 (2.197×)	**0.084 (29.878×)**	1.758 (1.427×)	0.325 (7.73×)	0.541 (4.64×)	0.87 (2.884×)
6000	1.301 (2.616×)	0.272 (12.494×)	3.404 (1.0×)	1.705 (1.997×)	**0.114 (29.862×)**	2.597 (1.311×)	0.513 (6.631×)	0.702 (4.851×)	0.848 (4.012×)
7000	1.693 (2.73×)	0.36 (12.825×)	4.622 (1.0×)	2.339 (1.976×)	**0.149 (31.018×)**	3.482 (1.327×)	0.72 (6.418×)	0.901 (5.127×)	0.802 (5.76×)
8000	2.305 (2.58×)	0.463 (12.853×)	5.946 (1.0×)	3.086 (1.927×)	**0.189 (31.46×)**	4.614 (1.289×)	0.992 (5.991×)	1.129 (5.267×)	0.819 (7.263×)
9000	2.866 (2.701×)	0.573 (13.505×)	7.742 (1.0×)	3.804 (2.035×)	**0.223 (34.719×)**	5.879 (1.317×)	1.175 (6.592×)	1.397 (5.543×)	0.806 (9.608×)
10000	3.612 (2.596×)	0.684 (13.701×)	9.377 (1.0×)	4.674 (2.006×)	**0.278 (33.729×)**	7.038 (1.332×)	1.481 (6.329×)	1.722 (5.445×)	1.014 (9.247×)
11000	—	—	11.629 (1.0×)	5.643 (2.061×)	**0.33 (35.239×)**	8.498 (1.368×)	1.817 (6.401×)	2.072 (5.613×)	1.021 (11.388×)
12000	—	—	13.612 (1.0×)	6.78 (2.008×)	**0.407 (33.444×)**	—	2.343 (5.81×)	2.45 (5.556×)	0.994 (13.693×)
13000	—	—	15.725 (1.0×)	7.924 (1.984×)	—	—	2.583 (6.087×)	2.754 (5.711×)	**1.068 (14.729×)**
14000	—	—	18.714 (1.0×)	9.758 (1.918×)	—	—	3.049 (6.138×)	3.264 (5.733×)	**0.947 (19.758×)**
15000	—	—	21.142 (1.0×)	10.657 (1.984×)	—	—	3.457 (6.116×)	3.711 (5.698×)	**0.991 (21.332×)**

Table S12. Simulation times (seconds) of different simulations for the network of **low-firing Izhikevich** neurons with respect to the network size (single run). The ratio of how much faster the simulator is over the slowest one with the same network size is shown in parentheses. The fastest simulator with respect to the network size is highlighted in bold. The empty cells represent instances where the system ran out of memory.

Network size	Simulators								
	ANNarchy CPU	ANNarchy GPU	Brian 2	Brian 2 C++	Brian 2 GPU	NEST	PymoNNto	PymoNNtorch CPU	PymoNNtorch GPU
10	0.002 (360.03×)	0.005 (185.687×)	0.381 (2.356×)	**0.002 (496.833×)**	0.009 (99.753×)	0.005 (189.319×)	0.024 (37.086×)	0.121 (7.445×)	0.898 (1.0×)
20	0.003 (294.487×)	0.005 (146.225×)	0.385 (1.939×)	**0.002 (367.089×)**	0.01 (74.592×)	0.005 (150.292×)	0.026 (28.666×)	0.143 (5.232×)	0.746 (1.0×)
50	0.003 (224.4×)	0.007 (114.442×)	0.387 (1.986×)	**0.003 (266.67×)**	0.01 (76.881×)	0.006 (131.908×)	0.026 (30.13×)	0.144 (5.356×)	0.769 (1.0×)
100	0.005 (159.211×)	0.008 (103.522×)	0.387 (2.09×)	**0.005 (176.575×)**	0.012 (67.481×)	0.008 (102.726×)	0.026 (31.054×)	0.151 (5.371×)	0.81 (1.0×)
250	0.013 (59.086×)	**0.011 (69.977×)**	0.394 (1.941×)	0.014 (54.248×)	0.013 (58.884×)	0.02 (37.671×)	0.031 (24.477×)	0.17 (4.502×)	0.765 (1.0×)
500	0.038 (24.893×)	**0.019 (48.67×)**	0.436 (2.155×)	0.047 (19.864×)	0.02 (47.023×)	0.06 (15.717×)	0.042 (22.568×)	0.195 (4.82×)	0.94 (1.0×)
750	0.086 (9.123×)	0.029 (26.65×)	0.511 (1.532×)	0.114 (6.862×)	**0.029 (26.98×)**	0.124 (6.287×)	0.057 (13.682×)	0.22 (3.56×)	0.782 (1.0×)
1000	0.157 (5.308×)	**0.039 (21.368×)**	0.618 (1.348×)	0.2 (4.167×)	0.042 (19.819×)	0.21 (3.962×)	0.082 (10.164×)	0.247 (3.371×)	0.832 (1.0×)
1500	0.452 (2.319×)	0.07 (14.959×)	1.05 (1.0×)	0.624 (1.681×)	**0.067 (15.665×)**	0.466 (2.254×)	0.138 (7.612×)	0.329 (3.187×)	0.78 (1.345×)
2000	0.924 (1.995×)	0.111 (16.556×)	1.842 (1.0×)	1.301 (1.416×)	**0.105 (17.544×)**	0.842 (2.188×)	0.217 (8.472×)	0.434 (4.241×)	0.924 (1.993×)
3000	2.289 (1.758×)	0.24 (16.775×)	4.025 (1.0×)	3.263 (1.233×)	**0.22 (18.295×)**	1.993 (2.02×)	0.697 (5.776×)	0.905 (4.449×)	0.805 (5.0×)
4000	4.415 (1.605×)	0.41 (17.298×)	7.085 (1.0×)	6.296 (1.125×)	**0.367 (19.305×)**	3.553 (1.994×)	1.673 (4.235×)	1.805 (3.925×)	0.9 (7.875×)
5000	7.211 (1.591×)	0.66 (17.396×)	11.474 (1.0×)	10.129 (1.133×)	**0.58 (19.783×)**	5.713 (2.008×)	2.84 (4.04×)	2.816 (4.075×)	0.878 (13.061×)
6000	10.475 (1.556×)	0.976 (16.697×)	16.303 (1.0×)	14.876 (1.096×)	**0.836 (19.501×)**	8.349 (1.953×)	4.597 (3.547×)	4.147 (3.932×)	1.046 (15.591×)
7000	14.543 (1.571×)	1.388 (16.458×)	22.844 (1.0×)	20.572 (1.11×)	1.139 (20.057×)	11.028 (2.072×)	6.47 (3.531×)	5.463 (4.182×)	**1.104 (20.683×)**
8000	19.386 (1.569×)	1.873 (16.24×)	30.418 (1.0×)	27.392 (1.11×)	1.482 (20.525×)	14.519 (2.095×)	9.034 (3.367×)	7.225 (4.21×)	**1.158 (26.264×)**
9000	24.609 (1.562×)	2.418 (15.894×)	38.434 (1.0×)	35.025 (1.097×)	1.874 (20.509×)	19.076 (2.015×)	10.411 (3.692×)	9.149 (4.201×)	**1.264 (30.408×)**
10000	30.353 (1.535×)	3.096 (15.046×)	46.588 (1.0×)	42.575 (1.094×)	2.329 (20.003×)	23.153 (2.012×)	12.472 (3.735×)	11.183 (4.166×)	**1.535 (30.346×)**
11000	—	—	57.181 (1.0×)	52.09 (1.098×)	2.854 (20.035×)	27.681 (2.066×)	15.585 (3.669×)	13.963 (4.095×)	**1.519 (37.635×)**
12000	—	—	68.843 (1.0×)	62.318 (1.105×)	3.347 (20.568×)	—	19.903 (3.459×)	16.367 (4.206×)	**1.741 (39.553×)**
13000	—	—	80.397 (1.0×)	73.208 (1.098×)	—	—	22.184 (3.624×)	18.708 (4.297×)	**2.129 (37.76×)**
14000	—	—	93.951 (1.0×)	85.293 (1.102×)	—	—	25.975 (3.617×)	22.482 (4.179×)	**2.046 (45.926×)**
15000	—	—	107.909 (1.0×)	99.671 (1.083×)	—	—	31.03 (3.478×)	25.064 (4.305×)	**2.263 (47.685×)**

Table S13. Simulation times (seconds) of different simulations for the network of **medium-firing Izhikevich** neurons with respect to the network size (single run). The ratio of how much faster the simulator is over the slowest one with the same network size is shown in parentheses. The fastest simulator with respect to the network size is highlighted in bold. The empty cells represent instances where the system ran out of memory.

Network size	Simulators								
	ANNarchy CPU	ANNarchy GPU	Brian 2	Brian 2 C++	Brian 2 GPU	NEST	PymoNNto	PymoNNtorch CPU	PymoNNtorch GPU
10	0.002 (335.518×)	0.005 (154.632×)	0.386 (2.099×)	**0.002 (436.6×)**	0.009 (89.94×)	0.005 (168.066×)	0.024 (33.713×)	0.126 (6.413×)	0.809 (1.0×)
20	0.003 (352.812×)	0.006 (165.197×)	0.384 (2.461×)	**0.002 (455.684×)**	0.01 (94.463×)	0.005 (189.737×)	0.026 (36.838×)	0.144 (6.574×)	0.945 (1.0×)
50	0.004 (230.419×)	0.008 (116.42×)	0.385 (2.283×)	**0.003 (284.174×)**	0.01 (87.98×)	0.006 (144.316×)	0.027 (33.149×)	0.152 (5.783×)	0.88 (1.0×)
100	0.006 (120.249×)	0.009 (86.29×)	0.388 (1.978×)	**0.005 (143.831×)**	0.013 (59.048×)	0.009 (80.953×)	0.028 (27.203×)	0.158 (4.864×)	0.768 (1.0×)
250	0.018 (45.969×)	**0.012 (71.045×)**	0.404 (2.09×)	0.019 (43.531×)	0.013 (64.975×)	0.028 (29.793×)	0.033 (25.628×)	0.177 (4.768×)	0.845 (1.0×)
500	0.061 (14.077×)	0.022 (39.414×)	0.461 (1.872×)	0.073 (11.884×)	**0.021 (41.136×)**	0.089 (9.668×)	0.047 (18.521×)	0.206 (4.19×)	0.864 (1.0×)
750	0.139 (5.49×)	**0.033 (23.26×)**	0.58 (1.312×)	0.185 (4.111×)	0.036 (21.122×)	0.19 (4.003×)	0.075 (10.153×)	0.244 (3.115×)	0.76 (1.0×)
1000	0.246 (3.421×)	**0.047 (18.096×)**	0.742 (1.136×)	0.328 (2.565×)	0.055 (15.32×)	0.33 (2.556×)	0.113 (7.48×)	0.29 (2.901×)	0.843 (1.0×)
1500	0.776 (1.901×)	**0.091 (16.198×)**	1.476 (1.0×)	1.068 (1.382×)	0.098 (15.059×)	0.723 (2.041×)	0.206 (7.159×)	0.401 (3.68×)	0.783 (1.884×)
2000	1.617 (1.665×)	**0.154 (17.478×)**	2.692 (1.0×)	2.348 (1.147×)	0.16 (16.828×)	1.323 (2.035×)	0.329 (8.184×)	0.559 (4.814×)	0.876 (3.072×)
3000	3.871 (1.626×)	**0.343 (18.351×)**	6.295 (1.0×)	5.569 (1.13×)	0.358 (17.582×)	3.195 (1.97×)	1.375 (4.579×)	1.436 (4.384×)	0.901 (6.985×)
4000	7.685 (1.506×)	0.631 (18.354×)	11.575 (1.0×)	10.873 (1.065×)	**0.611 (18.944×)**	5.781 (2.002×)	3.255 (3.556×)	2.841 (4.074×)	1.025 (11.291×)
5000	12.257 (1.512×)	1.074 (17.251×)	18.531 (1.0×)	17.636 (1.051×)	**0.975 (19.007×)**	9.035 (2.051×)	5.615 (3.3×)	4.461 (4.154×)	1.115 (16.616×)
6000	18.175 (1.484×)	1.663 (16.212×)	26.965 (1.0×)	25.896 (1.041×)	1.418 (19.016×)	13.118 (2.056×)	8.336 (3.235×)	6.542 (4.122×)	**1.114 (24.208×)**
7000	25.22 (1.505×)	2.415 (15.718×)	37.966 (1.0×)	35.723 (1.063×)	1.935 (19.621×)	17.546 (2.164×)	11.056 (3.434×)	8.654 (4.387×)	**1.214 (31.279×)**
8000	33.154 (1.524×)	3.301 (15.306×)	50.518 (1.0×)	47.807 (1.057×)	2.531 (19.96×)	23.244 (2.173×)	16.318 (3.096×)	11.366 (4.445×)	**1.561 (32.372×)**
9000	42.36 (1.498×)	4.371 (14.513×)	63.438 (1.0×)	60.433 (1.05×)	3.218 (19.713×)	30.188 (2.101×)	19.013 (3.336×)	14.365 (4.416×)	**1.613 (39.331×)**
10000	52.277 (1.497×)	5.689 (13.758×)	78.267 (1.0×)	73.472 (1.065×)	3.974 (19.695×)	37.02 (2.114×)	22.079 (3.545×)	18.108 (4.322×)	**1.78 (43.964×)**
11000	—	—	95.579 (1.0×)	90.304 (1.058×)	4.869 (19.63×)	44.398 (2.153×)	27.836 (3.434×)	20.887 (4.576×)	**1.978 (48.332×)**
12000	—	—	114.842 (1.0×)	109.363 (1.05×)	5.742 (20.0×)	—	36.146 (3.177×)	25.967 (4.423×)	**2.261 (50.797×)**
13000	—	—	132.648 (1.037×)	137.547 (1.0×)	—	—	39.712 (3.464×)	29.998 (4.585×)	**2.7 (50.939×)**
14000	—	—	155.124 (1.0×)	146.779 (1.057×)	—	—	45.066 (3.442×)	36.655 (4.232×)	**2.896 (53.573×)**
15000	—	—	176.087 (1.0×)	169.253 (1.04×)	—	—	54.785 (3.214×)	40.434 (4.355×)	**3.066 (57.424×)**

Table S14. Simulation times (seconds) of different simulations for the network of **high-firing Izhikevich** neurons with respect to the network size (single run). The ratio of how much faster the simulator is over the slowest one with the same network size is shown in parentheses. The fastest simulator with respect to the network size is highlighted in bold. The empty cells represent instances where the system ran out of memory.

REFERENCES

Pauli, R., Weidel, P., Kunkel, S., and Morrison, A. (2018). Reproducing polychronization: A guide to maximizing the reproducibility of spiking network models. *Frontiers in Neuroinformatics* 12. doi:10.3389/fninf.2018.00046
